# High-throughput discovery of post-transcriptional *cis*-regulatory elements

**DOI:** 10.1186/s12864-016-2479-7

**Published:** 2016-03-03

**Authors:** Erin M. Wissink, Elizabeth A. Fogarty, Andrew Grimson

**Affiliations:** Department of Molecular Biology and Genetics, Cornell University, 445 Biotech, Ithaca, NY 14853 USA; Present address: Department of Molecular, Cell, and Developmental Biology, University of California at Los Angeles, Los Angeles, CA 90095 USA

**Keywords:** Post-transcriptional gene regulation, 3′UTR, High-throughput screen, mRNA decay, Regulatory element

## Abstract

**Background:**

Post-transcriptional gene regulation controls the amount of protein produced from an individual mRNA by altering rates of decay and translation. Many sequence elements that direct post-transcriptional regulation have been found; in mammals, most such elements are located within the 3′ untranslated regions (3′UTRs). Comparative genomic studies demonstrate that mammalian 3′UTRs contain extensive conserved sequence tracts, yet only a small fraction corresponds to recognized elements, implying that many additional novel elements exist. Despite a variety of computational, molecular, and biochemical approaches, identifying functional 3′UTRs elements remains difficult.

**Results:**

We created a high-throughput cell-based screen that enables identification of functional post-transcriptional 3′UTR regulatory elements. Our system exploits integrated single-copy reporters, which are expressed and processed as endogenous genes. We screened many thousands of short random sequences for their regulatory potential. Control sequences with known effects were captured effectively using our approach, establishing that our methodology was robust. We found hundreds of functional sequences, which we validated in traditional reporter assays, including verifying their regulatory impact in native sequence contexts. Although 3′UTRs are typically considered repressive, most of the functional elements were activating, including ones that were preferentially conserved. Additionally, we adapted our screening approach to examine the effect of elements on RNA abundance, revealing that most elements act by altering mRNA stability.

**Conclusions:**

We developed and used a high-throughput approach to discover hundreds of post-transcriptional *cis*-regulatory elements. These results imply that most human 3′UTRs contain many previously unrecognized *cis*-regulatory elements, many of which are activating, and that the post-transcriptional fate of an mRNA is largely due to the actions of many individual *cis*-regulatory elements within its 3′UTR.

**Electronic supplementary material:**

The online version of this article (doi:10.1186/s12864-016-2479-7) contains supplementary material, which is available to authorized users.

## Background

Post-transcriptional regulatory events govern both the rates of mRNA decay and translation, thus controlling the amount of time an mRNA can productively interact with ribosomes [1]. In mammals, post-transcriptional regulation is primarily encoded by short *cis*-regulatory elements located in an mRNA’s 3′ untranslated region (3′UTR) [2]. Because human 3′UTRs have an average length of ~1,300 nucleotides, an individual 3′UTR has the potential to contain many elements [3]. Moreover, comparative genomic studies indicate that a large proportion of 3′UTR sequence is under selection, and these conserved regions likely correspond to regulatory elements [4, 5]. Taken together, it is likely that most 3′UTRs include multiple regulatory sequences, the majority of which remain to be described. Identifying which sequences have functional roles, and the mechanisms by which those sequences act, is required to understand the biology of 3′UTRs and the post-transcriptional regulation they mediate.

The predominant *trans*-factors that interact with 3′UTR *cis*-regulatory elements are microRNAs (miRNAs) and mRNA binding proteins (mRBPs). MicroRNAs are thought to have consequential target sites in most human genes [6], many of which contain multiple target sites [7]. Additionally, 3′UTRs are known to be extensively bound by a wide variety of mRBPs [8–10], indicating that transcripts likely contain many discrete post-transcriptional *cis*-regulatory elements, a conclusion corroborated by detailed studies of individual 3′UTRs [11–14]. Importantly, because miRNA binding within a 3′UTR derives, predominantly, from base-pairing interactions, the systematic identification of miRNA target sites is somewhat straightforward [15]. In contrast, mRBP recognition of primary sequence and secondary structure in mRNAs relies upon each individual protein’s structure and sequence, which have far more diverse biochemical properties than different nucleic acid sequences in small regulatory RNAs [16–19]; thus, systematic identification of binding sites for even a single RBP requires extensive empirical testing. Importantly, although the preferred binding sites for both miRNAs and a subset of mRBPs are known [20, 21], determining which in vivo sites are functional remains a major challenge.

Fluorescence-based screens have been used in a variety of contexts to study gene regulation, including discovery of splice enhancers [22], the impact of codon choice on expression [23], and identification of DNA enhancer elements [24]. Recent work has extended the use of fluorescence-based screens to identify sections of endogenous genes that regulate post-transcriptional gene expression [12, 25]. These previous studies focused upon relatively large sections of 3′UTRs that likely contained multiple regulatory elements, thus compromising the ability to attribute regulatory impact to discrete elements. Nevertheless, it is clear that high-throughput assays facilitate the efficient interrogation and identification of sequences that function in post-transcriptional gene regulation or other regulatory steps.

Here, we describe a novel high-throughput screen designed to identify individual 3′UTR-encoded functional sequences that direct post-transcriptional regulation. Our system exploits integrated dual-fluorophore reporter libraries, in conjunction with fluorescence activated cell sorting (FACS), to enrich for cells containing functional sequences within the library. Functional elements are then identified using high-throughput sequencing. Using this approach, we discovered hundreds of candidate *cis*-regulatory elements, many with no known *trans*-acting binding partner. This work provides a powerful new tool to continue to interrogate regulatory information within 3′UTRs, and demonstrates that a multitude of *cis*-regulatory elements within 3′UTRs remain to be characterized.

## Results

### A system for measuring the regulatory impact of many 3′UTR sequences in parallel

**Fig. 1:**
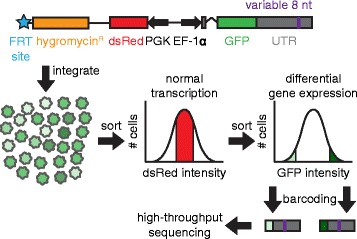
Experimental design. GFP reporter expression was driven by the EF-1α promoter and potentially modulated by a variable 8mer inserted into the human *IQGAP1* 3′UTR. The 5′UTR of the GFP reporter contains an intron. Expression of dsRed was used to control for transcriptional noise at the reporter locus, and was driven by the PGK promoter. The flippase recombination target (FRT) site allows this plasmid to undergo site-specific recombination in HEK293-TRex-FLP cells, such that only cells that integrate this construct at the intended locus via the FRT site gain hygromycin resistance. After integration, cells with normal transcriptional activity at the reporter locus (as determined by dsRed levels), and that are potentially undergoing differential post-transcriptional regulation (as determined by GFP levels), were isolated via fluorescence activated cell sorting (FACS). From FACS isolated sub-populations, the portion of the 3′UTR containing the variable 8mer was PCR amplified, thereby adding Illumina adapter sequences, and allowing 8mers in each sorted population to be identified and quantified by Illumina sequencing

The post-transcriptional fate of mammalian genes is primarily regulated by sequence elements located within mRNA 3′UTRs. Here, we developed and used a cell-based assay in which the expression of a GFP fluorescent-reporter library, coupled with high-throughput sequencing, acted as a readout for the regulatory potential of short sequences inserted within a reporter 3′UTR (Fig. 1, Additional file 1). These reporter construct libraries were integrated in parallel as a pool into the genomes of HEK293-FLP cells at a single locus, such that each individual cell received only a single member of the library. After selecting for successful reporter integration, cells exhibiting differential GFP expression, as compared to the overall population of GFP+ cells, were isolated by fluorescence activated cell sorting (FACS). High-throughput sequencing was used to identify the proportion of cells containing each different reporter construct in both the overall cell population and in sub-populations defined by GFP intensity. Sequences enriched within GFP_high_ populations corresponded to candidate activating elements, whereas sequences enriched in GFP_low_ populations were candidate repressive elements.

Our screening system was designed to recapitulate endogenous gene structure and expression, incorporating multiple features that enabled the reporter gene to undergo normal mRNA synthesis and processing. First, unlike previous high-throughput screens used to test the efficacy of potential post-transcriptional regulatory elements [12, 25], we inserted short random sequences to be assayed within the human *IQGAP1* 3′UTR, thus ensuring that the candidates we identified would be functional within an endogenous 3′UTR sequence. Moreover, we established that exogenous regulatory sequences were capable of mediating regulation at the insertion position by demonstrating that an inserted microRNA target site added there elicited the level of repression expected in response to the cognate miRNA (Additional file 1). Second, by using the FLP-FRT technology, the reporter integrated as a single copy at a defined locus, thus improving the signal-to-noise ratio by removing the impact of the integration site on expression. Third, because most human genes contain introns [26] and because splicing facilitates subsequent steps in an mRNA’s life cycle including export and translation [27, 28], we included an intron within our reporter gene. Importantly, while developing our screening strategy, we found that site-specific integration using FLP-FRT technology occasionally resulted in stochastic yet heritable changes in reporter gene expression. To solve this problem, we co-integrated a second fluorescent reporter, dsRed, together with our GFP reporter. Measuring dsRed expression, therefore, allowed us to greatly improve the performance of our system by excluding cells undergoing differential transcription at the reporter locus (Additional file 1). Together, these features provided a stringent manner in which to test the post-transcriptional regulatory potential for thousands of sequences in parallel.

### Discovering novel 3′UTR *cis*-regulatory elements

To discover regulatory sequences in a global and unbiased manner, we first performed a pilot screen, testing a large library of random 8-nucleotide sequences (8mers) inserted within the *IQGAP1* 3′UTR (Additional file 2). We chose 8mers because they are small enough to likely contain at most one regulatory sequence, yet large enough to interact specifically with many mRBPs [19]. Although this screen identified ~1,100 putative regulatory elements from the ~8,000 assayed (Additional files 2 and 3), we estimated a false positive error rate of ~50 %, as determined by testing candidate regulatory elements in luciferase reporter assays (Additional file 2). To more reliably identify functional 3′UTR regulatory elements, we performed a more focused screen in which we included candidates from the pilot screen and other possibly interesting motifs, such as previously identified mRBP binding motifs, together with negative control sequences (Additional file 4). Importantly, this focused screen incorporated several enhancements to our original approach: (i) we tested fewer elements (3,918), thus increasing the number of independent integration events per tested element; (ii) we sorted a stringent background set of cells, allowing us to determine which 8mers were robustly included in the experiment, (iii) we analyzed populations of cells that span the GFP distribution, rather than the tails of the distribution alone, allowing us to monitor the entire range of post-transcriptional regulation; and (iv) we sorted more cells (≥5x10^5^) per sorted sub-population, providing more replicates per sort. Together, these enhancements allowed us to reduce screening noise and produce a robust measure of regulatory potential for each tested 8mer.

**Fig. 2:**
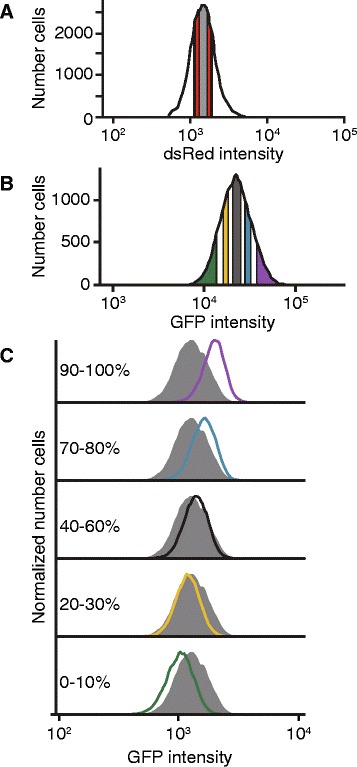
Isolating cells undergoing differential gene regulation. **a** dsRed intensity was measured in cells expressing the reporter construct shown in Fig. 1. The cells with the middle 50 % of dsRed intensity (shown in red and gray) were used for subsequent steps; GFP+ cells with the middle 25 % of dsRed intensity (shown in gray) were also collected as a stringent background set. **b** Five GFP-positive sub-populations were sorted from the red/gray population in A: 0–10 % (green; lowest GFP bin), 20–30 % (yellow), 40–60 % (gray), 70–80 % (blue), and 90–100 % (purple; highest GFP bin). For each, 5x10^5^ cells were collected, in duplicate. Additionally, 10^6^ GFP+ cells were sorted and retained (GFP-ALL). **c** Sorted cells had heritable differences in GFP expression. Three weeks after sorting, the populations’ fluorescence were measured via FACS. The GFP intensities for the indicated GFP-sub populations (color-coded to match panel B) are shown overlaying the GFP intensities for the GFP-ALL population; the number of cells were normalized to the mode GFP intensity

We generated >3 × 10^5^ cells that underwent independent site-specific integration events from members of our reporter library (Additional file 5), corresponding to an average of ~8 independent integration events per motif tested. We used FACS to sort all GFP+ cells that fell in a 50 % dsRed cutoff (25th-75th percentile of intensity) as one background set. To generate a second, more stringent background set, we also sorted the GFP+ cells falling the in the middle 25 % of dsRed + cells (37.5–62.5 percentile). In our later analyses, we only included 8mers found in this narrow population, reasoning that such cells had normal transcriptional activity at the reporter locus (Fig. 2a). We next sorted five cell populations that spanned the range of GFP intensity: 0–10, 20–30, 40–60, 70–80, and 90–100 % (Fig. 2b), corresponding to a range of intensities ordered from lowest to highest, and isolating only cells that fell within the middle 50 % dsRed cutoff (Fig. 2b, Additional file 6). Each population was sorted in duplicate, and replicates were maintained separately in all subsequent steps. Importantly, three weeks after sorting, the GFP-subset populations exhibited stable changes in GFP intensity that corresponded to the GFP intensities of the cells when they were initially sorted (Fig. 2c); replicate sorted populations had concordant GFP intensities (Additional file 7). As expected, different GFP populations had near identical dsRed expression post-sorting (Additional file 7). These results demonstrate that changes in GFP intensity are heritable and that populations of cells can be isolated that have differing reporter gene regulation.

### Previously identified regulatory elements are enriched in expected cells populations

Reporters with altered GFP expression contain 8mers that alter gene regulation, thus the GFP bin in which an 8mer is enriched predicts the strength of the 8mer’s regulatory effect. To quantify 8mer enrichment in the sorted populations, we isolated DNA from all GFP+ cells and from each GFP sub-population. We then amplified the segment of the *IQGAP1* 3′UTR containing the 8mers, using a PCR strategy that appended sequences appropriate for multiplexed Illumina sequencing (Fig. 1, Additional file 1). High-throughput sequencing data was obtained for all sorted cell populations. The 8mer counts correlated well between replicate sorting populations (Pearson *r* > 0.97; *p* < 10^-15^); we used the replicate sorting data to determine which 8mers were robustly included in the background set (Additional file 8). We found the proportion of each robustly included 8mer in each GFP+ sub-populations, scaled by its overall abundance in the library (Additional file 9). Thus, we could determine if an 8mer was enriched or depleted across all GFP+ sub-populations and thereby infer whether the motif was activating, repressive, or had no regulatory effect.

**Fig. 3:**
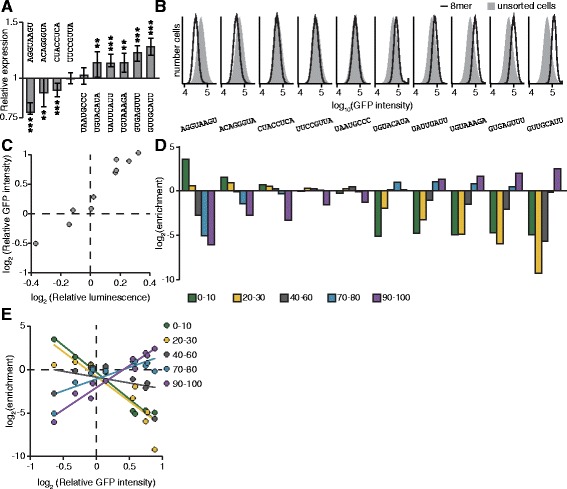
Assaying 8mers with known regulatory properties. **a** Regulatory effects of expression controls. Each control sequence was inserted into the *IQGAP1* 3′UTR within a luciferase reporter. The luciferase activity of these reporter constructs were normalized to the geometric means of two control sequences with no regulatory effect (UUCCGUUA and UAAUGCCC). The normalized geometric means are plotted ± 33 % of the spread of the data. Significance was determined by two-sided Wilcoxon rank sum tests; *n* = 9. * *p* < 0.05, ** *p* < 0.005, *** *p* < 0.0005. **b** Expression of control sequences when inserted into the integrated GFP reporter. The distribution of GFP intensity is shown for each control as compared to unsorted cells. **c** Correlation of relative luminescence and GFP values. The geometric mean of the GFP intensities for each expression control were calculated and normalized to the geometric means of two control sequences with no regulatory effect (UUCCGUUA and UAAUGCCC). These values (y-axis) were plotted against the relative luminescence values (x-axis) found in B. Pearson *r* = 0.975, *p* < 10^−5^. **d** Performance of expression controls in the main screen. Cells containing each of the ten expression controls were added to the library of cells prior to sorting. DNA was isolated from the sorted populations, and the 8mers were identified and quantified. Read values were normalized by the number of reads in each sequencing library (reads per million, RPM). The enrichment of each expression control was calculated by dividing the RPM values in each GFP sub-population by the RPM value in GFP-ALL cells. **e** Correlating enrichment in sorting bins with an 8mer’s GFP intensity. Each control’s relative GFP intensity (x-axis) is plotted against enrichment in the five sorting bins (y-axis), and the lines of best fit are shown. Correlation values (Pearson r) for each bin against intensity are as follows: 0–10 % (*r* = −0.978, *p* < 10^−5^); 20–30 % (*r* = −0.863, *p* < 0.005); 40–60 % (*r* = 0.369, not significant); 70–80 % (*r* = 0.787, *p* < 0.05); 90–100 % (*r* = 0.972, *p* < 10^−5^)

We designed the focused screen to include ten internal control 8mers, whose regulatory effects we had determined previously. Five of the ten corresponded to established post-transcriptional *cis*-regulatory elements; the remaining five were novel elements identified from our pilot screen (Additional files 2 and 3), which we had subsequently validated (Table 1). The ten sequences were selected to represent a range of impacts on gene expression; three were repressive, two had no effect in the 3′UTR sequence context used here, and five were activating (Fig. 3a-c). We determined their regulatory impacts both using luciferase assays (Fig. 3a), and as individual integrated 8mers assayed using our GFP reporter system (Fig. 3b). Importantly, the regulatory impacts of the ten sequences correlated strongly between transient transfection luciferase assays and integrated GFP reporter measurements (Fig. 3c, Pearson *r* > 0.97; *p* < 10^−5^).

**Table 1:** Sequences used as internal controls

Sequence	Effect	Role
AGGUAAGU	Repressive	Novel
ACAGGGUA	Repressive	miR-10 target site
CUACCUCA	Repressive	let-7 target site
UUCCGUUA	No effect	miR-191 target site
UAAUGCCC	No effect	Novel
UGUACAUA	Activating	Pumilio binding motif
UAUUUAUU	Activating	AU rich element
UGUAAAGA	Activating	Novel
GUGAGUUU	Activating	Novel
GUUGCAUU	Activating	Novel

The level of enrichment of the ten control 8mers in the sorted GFP sub-populations reflected their individually determined activities in reporter assays (Fig. 3d), indicating the quantitative nature of the screen. For example, AGGUAAGU, which is the most repressive control element, is highly enriched in the lowest GFP intensity bin (the 0–10 % bin), slightly enriched in the 20–30 % bin, and depleted in the other bins. GUUGCAUU, which is the most activating control element, had the converse enrichment pattern: it is strongly enriched in the highest GFP intensity bin (the 90–100 % bin), at background level in the 70–80 % bin, and depleted in the other bins. Elements with no effect were near background level in all bins. Importantly, there was near-perfect concordance between the reporter data and the screen data across the ten elements (Fig. 3d). Overall, the enrichment in the low GFP bins (0–10 and 20–30 %) negatively correlated with an element’s GFP intensity, whereas the enrichment in the high GFP bins (70–80 and 90–100 %) positively correlated with GFP intensity (Fig. 3e), suggesting that a comprehensive score for each 8mer that incorporated the enrichment values across all five bins would provide a semi-quantitative prediction of regulatory impact. Together, these data demonstrate that known sequences have the expected performance in this screen, implying that the regulatory effect of novel sequences could also be quantified.

### Discovering novel post-transcriptional *cis*-regulatory elements

**Fig. 4:**
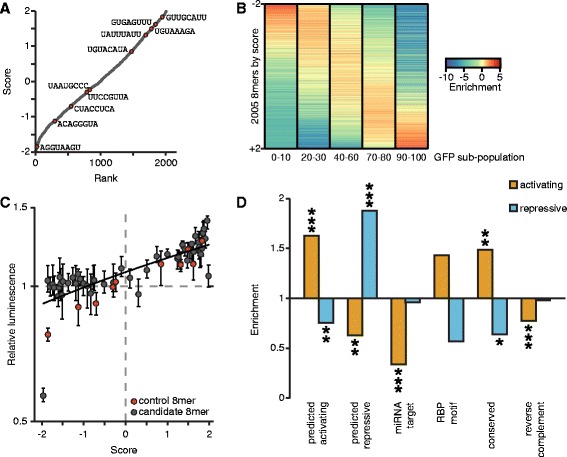
Identifying 8mers with regulatory potential. **a** Scoring all tested 8mers. An expression score was calculated for each 8mer by multiplying the RPM values in each GFP sub-population by a scaling factor (0–10 %: −2, 20–30 %: −1, 40–60 %: 0, 70–80 %: 1; 90–100 %: 2), then normalized by the summed RPM value. Each 8mer is plotted by rank (x-axis) and score (y-axis). The expression controls are marked in red. **b** The enrichment of each 8mer in each GFP sub-population was found by dividing its RPM values by the RPM values in GFP+ cells. The enrichment values are ordered by the expression score shown in A. **c** Candidate elements recapitulate their behavior observed in the primary screen. Each candidate was inserted into the *IQGAP1* 3′UTR-luciferase construct, and the luciferase activity of these reporter constructs were normalized to the geometric means of two expression controls with no regulatory effect (UUCCGUUA and UAAUGCCC). The relative luminescence values (y-axis) were plotted versus the expression score (x-axis) for each candidate sequence. Expression controls are indicated in red. Pearson *r* = 0.820, *p* < 10^−5^. **d** The 8mers with scores >1 were considered activating elements, and those scores < −1 were considered repressive elements. The enrichment for each category of sequences was determined for activating and repressive elements. Significance was assessed by two-sided Fisher exact tests; *p < 0.05, **p < 0.005, ***p < 0.0005

To quantify function for all sequences tested in our screen, we calculated a score for each element based on its sequencing counts in the five GFP-subset sorting populations. Each bin was given a weighted value (0–10 %:–2, 20–30 %: −1, 40–60 %: 0, 70–80 %: 1; 90–100 %: 2); as a result, repressive sequences had negative scores and activating sequences had positive scores, with all scores falling within the range of −2 to +2, corresponding to maximally repressive and maximally activating, respectively. The score and relative rank are shown for each sequence tested in the screen (Fig. 4a). The ten control elements, which are also shown, span the range of possible scores and correspond well with their regulatory effects on GFP intensity (Fig. 3c).

To assess the consistency of our scoring metric, we ordered the 8mers by their score, and examined the relationship between score and the enrichment values we observed in each of the five GFP+ sub-populations (Fig. 4b, Additional file 10). As expected, lowly ranked sequences are enriched in the low GFP bins and depleted in the high GFP bins, while highly ranked sequences have the reciprocal enrichment pattern. Sequences that are ranked in the middle are enriched in the 40–60 % sorting bin and depleted in both the very high and very low sorting bins, demonstrating that they likely have little to no effect on post-transcriptional regulation (Fig. 4b, Additional file 10).

To determine how well our screen identified novel regulatory motifs, we selected fifty 8mers with scores ranging from −1.965 to 1.986 to validate using luciferase reporter assays. For comparison, we also included the ten control 8mers in these experiments. There was good correlation between each sequence’s score from the screen data and its measured regulatory effect in the luciferase reporter assays (Fig. 4c), indicating that scores effectively predict an element’s regulatory effect in an orthogonal reporter assay Additional file 11.

To determine the sensitivity of the screen, we investigated how the regulatory effects of tested 8mers corresponded to sequences known to mediate post-transcriptional regulation. We defined activating and repressing 8mers from our screen by using expression scores for the control elements. Amongst the control elements, the activating sequences UAUUUAUU, UGUAAAGA, GUGAGUUU, and GUUGCAUU all have scores greater than +1, whereas the repressive elements AGGUAAGU and ACAGGGUA have scores less than −1; thus, we used those score thresholds on the entire data set, resulting in 372 repressive elements and 461 activating elements (listed in Additional file 9). We observed consistency between our original screen and the focused screen. Encouragingly, 8mers that we had previously predicted to be activating were significantly enriched in activating elements in our main screen, and they were depleted from repressive 8mers. Similarly, the 8mers that we had previously predicted to be repressive had significant enrichment in elements defined as repressive in the main screen, and they were depleted from activating 8mers (Fig. 4d).

We next examined specific subclasses of known *cis*-regulatory elements, including miRNA target sites and RBP binding sites, together with conserved motifs. Perhaps surprisingly, miRNA target sites (Additional file 12) were not significantly enriched in repressive sites. This lack of signal may reflect the absence of the cognate miRNA in the cells we used and/or the relatively subtle effect miRNAs mediate. As determined by miRNA profiling in HEK293-FLP cells, the most abundant miRNA is miR-10, and the 8mer corresponding to the miR-10 target site was found to be repressive in our screen (ACAGGGUA, Fig. 3a). It is worth noting that we did find significant depletion of miRNA target sites within the set of elements predicted as activating. Our screen also included sequences corresponding to the binding sites for certain RNA binding proteins (Additional file 13); however, these did not show significant enrichment in either category. We also included elements we found to be frequently conserved within mammalian 3′UTRs, which we had anticipated would be repressive because 3′UTRs are generally considered negative regulatory sequences [29, 30]. These conserved sequences were instead enriched in the set of 8mers our screen predicts as activating sequences, suggesting that many functional post-transcriptional *cis*-regulatory motifs are positive regulatory sequences.

We repeated our enrichment tests across all categories using a range of cutoffs for active elements; importantly, our results were robust to analyses using these additional cutoffs (Additional file 14). Additionally, our data suggest that the majority of elements act on RNA at the post-transcriptional level because the scores of 8mers that are reverse complement pairs do not positively correlate (Additional file 15). Moreover, the reverse complements to elements predicted to be functional (including previously screened 8mers, miRNA target sites, RBP motifs, and conserved elements) are not enriched in activating or repressive sequences, demonstrating they are not functional (Fig. 4d).

### Sequence elements often affect mRNA stability

**Fig. 5:**
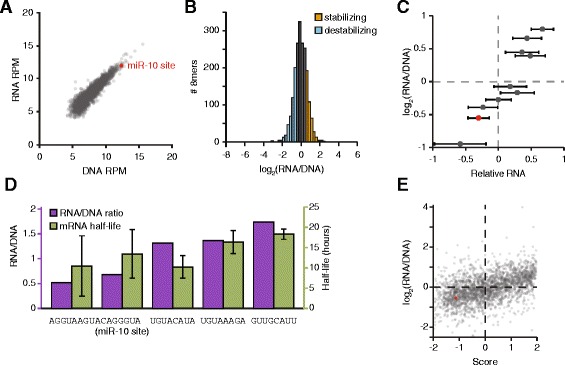
RNA levels for each sequence correlate to expression score. **a** The normalized read counts for each 8mer were found in DNA (x-axis) and RNA (y-axis) isolated from GFP-positive cells and gated within the middle 25 % of dsRed expression. The miR-10 target site is depicted in red. Pearson *r* = 0.916, *p* < 10^−15^. **b** Distribution of RNA/DNA ratios for all tested 8mers. 8mers with log_2_(RNA/DNA) > 0.5 are shown in orange, and < −0.5 in blue. **c** RNA abundance for ten control 8mers. RNA was isolated and quantified from ten cell lines in which expression control sequences were inserted into GFP and integrated in the genome. Shown is the mean of values normalized to that of UUCCGUUA. *n* = 2; error bars are propagated error from 3 technical replicates per biological replicate. Normalized RNA values (x-axis) for each 8mer are plotted versus the ratio of RNA/DNA determined in the screen. The miR-10 target site is depicted in red. Pearson *r* = 0.951, *p* < 10^4^. **d** Transcript half-lives correlate with RNA/DNA ratios. Cell lines in which individual 8mers were integrated were used to find transcript half-lives. Data from 3–6 replicates were combined to find half-lives, error bars indicate standard deviation. Pearson *r* = 0.675. **e** The expression score (x-axis) and the RNA/DNA ratio (y-axis) are shown for each 8mer. The miR-10 target site is depicted in red. Pearson *r* = 0.464, *p* < 10^−15^

Post-transcriptional regulation can control gene expression at multiple levels, including predominantly the control of mRNA stability and translation. We modified our system to gain insights into the mechanism by which the novel elements we discovered acted, in particular whether elements acted by regulating mRNA stability. Our sorting data provided protein expression information, whereas sequencing DNA from sorted cells provided information regarding the number of cells containing each 8mer. We next quantified each 8mer’s abundance in mRNA transcripts produced from the GFP reporter, creating a cDNA representation of our reporter library, in addition to the genomic DNA derived library (Fig. 1). This approach allowed us to quantify relative steady-state RNA levels for each reporter within our library and thereby identify elements that resulted in increased or decreased transcript abundances per cell. The level of each 8mer within the cDNA library correlated well to its level in genomic DNA isolated from the same cells (Fig. 5a, Additional file 9), suggesting that most individual 8mers we tested have relatively small effects on RNA abundance, as expected. To examine this relationship further, we normalized RNA abundances for each 8mer to its DNA abundance (Fig. 5b), then chose thresholds based on the RNA/DNA ratio for an 8mer with known effects on mRNA stability, the miR-10 target site. Because miRNAs increase the decay rate of their mRNA targets [31], this 8mer’s transcript is expected to be destabilized. The log_2_ ratio for ACAGGGUA is −0.55, and so we chose 0.5 and −0.5 as the cutoffs for log_2_(RNA/DNA) ratio score thresholds for elements that potentially alter mRNA stability ratios. Taking this approach, we found 457 8mers that destabilize their mRNAs (23 %) and 417 that stabilize them (21 %).

To confirm that our approach could reliably determine the relative RNA/DNA ratio for each 8mer, we tested our ten control sequences individually using qRT-PCR assays. The resulting values correlated well with our high-throughput RNA/DNA measurements (Fig. 5c), demonstrating that the RNA/DNA ratio is an appropriate measurement of steady-state RNA for each 8mer. Importantly, when we determined the half-lives for five of the mRNAs, these also correlated well with the RNA/DNA ratio (Fig. 5d, Additional file 16), indicating that differences in RNA steady-state levels are predominantly due to post-transcriptional regulation.

To explore if 8mers in our screen affected mRNA stability or translation rate, we compared the RNA/DNA ratio, which is a measure of differential RNA abundance, to the previously calculated expression scores (Fig. 4a), which reflect GFP protein abundance. These two values correlated significantly (Fig. 5e), implying that many sequences regulate protein expression by altering mRNA stability. Evidence of post-transcriptional regulation via translation rate is seen, however, in those sequences that exhibited low expression scores and high DNA/RNA ratios, and vice versa. One caveat of this approach is that it cannot distinguish between 8mers that affect stability alone from ones that affect both stability and translation. Together, these results demonstrate that high-throughput screens can be adapted to discern mechanistic details of post-transcriptional gene regulation.

### Predicted candidates regulate endogenous 3′UTRs

**Fig. 6:**
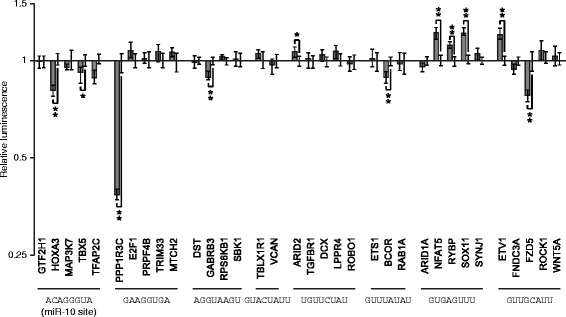
Motifs found in the screen regulate endogenous 3′UTRs. Human 3′UTRs containing conserved instances of motifs found from the screen were identified. Sequences from human, mouse, rat, and dog 3′UTRs were aligned, and 3′UTRs which contained an intact and orthologous instance of the 8mer in the four species were considered. Fragments of ~500 nt of human 3′UTRs containing conserved motifs were inserted into luciferase reporter constructs. The candidate motif was mutated at three positions to create a negative control, reasoning that three sequence changes were sufficient to ablate potential function of the original motif. The luciferase activity of constructs containing the intact motif were normalized to the construct with a mutated motif. Reporter data are plotted as the geometric mean of relative luminescence (y-axis) of reporter constructs normalized to those with mutated sites; error bars indicate 68 % of the data. Significance was determined by a two-sided Wilcoxon rank sum test, *n* = 6; *p < 0.05, **p < 0.005 after Bonferonni correction

The regulatory sequences discovered here were identified in the context of a reporter gene with a single human 3′UTR. To examine the regulatory impact of these sequences in endogenous contexts, we measured the regulatory impact of eight different 8mers in the context of human 3′UTRs in which they are naturally found. Three of the 8mers we selected were identified as repressive elements (ACAGGGUA, GAAGGUGA, and AGGUAAGU), and five as activating (GUACUAUU, UGUUCUAU, GUUUAUAU, GUGAGUUU and GUUGCAUU). ACAGGGUA, which is the target site of miR-10, a miRNA that is highly expressed in HEK-293 FLP cells (Additional file 12), was included within the eight as a control. For each, we created multiple luciferase reporter constructs containing ~500 nt of a human 3′UTR containing a conserved exemplar of the motif. We generated paired control reporters in which we mutated three of the nucleotides within the 8mer to inactivate the candidate element. The effect of each 8mer in each 3′UTR tested was found by comparing its luciferase levels to the corresponding control reporter (Fig. 6). Reporters monitoring miR-10 target sites demonstrated that this element was repressive, as expected, yet only two of the five tested 3′UTRs contained detectably functional miR-10 sites, confirming previous work showing that the sequence context surrounding *bona fide* regulatory elements determines their efficacy [15, 32]. Similarly, the two candidate repressive elements were validated as repressive in some, but not all, native 3′UTR contexts. Three of the tested activating elements (UGUUCUAU, GUGAGUUU, GUUGCAUU) were able to increase gene expression in certain 3′UTR contexts, with only the element GUGAGUUU functional as an activating sequence in most contexts examined. Interestingly, the sequence GUUGCAUU is able to both increase and decrease reporter activity, depending on the 3′UTR in which it is found. These results establish that candidate elements discovered from this screen have functional roles in endogenous genes, and are broadly comparable to miRNA target sites in terms of the degree of regulation they mediate. Moreover, our results highlight the importance of sequence context on the roles of individual sequence elements within 3′UTRs.

## Discussion

In this work, we identified hundreds of novel post-transcriptional *cis*-regulatory elements using a fluorescence-based, high-throughput, functional cell-based screen. We validated our screen data with orthogonal luciferase reporter assays and found strong agreement between the two readouts. We were able to assign a regulatory impact score to thousands of 8mers that predicted if each 8mer altered gene expression, and if so, if it was activating or repressive. Using this approach, we found 461 activating and 372 repressive elements. Interestingly, 8mers with preferential conservation in 3′UTRs were enriched in activating elements, suggesting that many functional post-transcriptional *cis*-regulatory elements act to increase gene expression. In a parallel complementary high-throughput screen, we established that the majority of functional 8mers we found acted by altering mRNA stability. Finally, we confirmed that a sample of 8mers identified in our screen were functional in native 3′UTR contexts, demonstrating that our method discovered *bona fide cis*-regulatory elements. These results, therefore, suggest that 3′UTRs contain many short *cis*-regulatory elements that together determine the post-transcriptional fate of an mRNA.

Our approach complements other related studies that also measured the effect of 3′UTR sequences on gene expression [12, 25]. The goal of these previous studies was to identify larger, and perhaps structured, elements within native sequence contexts; thus, the regulatory impacts of larger regions of 3′UTRs were assayed, focusing on conserved sequences. These studies provided important insights into post-transcriptional regulation but were not designed to determine the effects of individual regulatory elements. These approaches, however, are well suited to identifying regulatory elements that incorporate structured RNA. In contrast, our approach instead found individual *cis*-regulatory elements by measuring the effects of sequences that are short enough to interact with one protein domain that binds single-stranded RNA, such as an RNA recognition motif or KH domain [16, 17]. By focusing on such short sequences, we hoped to define discrete *cis*-regulatory elements, and indeed, we found hundreds of 8mers that exert regulatory effects and presumably interact with RNA binding proteins. Currently, the relative importance and frequency of short sequence elements versus structured 3′UTR elements in mediating post-transcriptional regulation is unknown. It is clear that further understanding of post-transcriptional regulatory sequences will benefit from combining multiple approaches, including functional screens, such as our system, with approaches such as CLIP-Seq that identify mRNA binding sites of RBPs.

Because post-transcriptional gene regulation is primarily encoded within 3′UTRs, it is important to determine how many *cis*-regulatory elements are present and functional in each 3′UTR. Most human genes include conserved target sites of multiple miRNAs [6, 7], but the data for motifs that interact with RBPs are less clear, due to lack of knowledge of their preferred binding sequences. Global CLIP studies have been used to identify all positions in mRNAs that cross-link to proteins [8–10]; however, these sites are often not functional [15]. It is thus difficult to determine how RNA binding proteins affect post-transcriptional regulation of individual 3′UTRs. Combining CLIP data with comparative genomics approaches is useful in that it allows partitioning by evolutionary conservation [33]. The data reported here, as well in other studies that have performed functional experiments in 3′UTRs [11, 12, 14, 25], demonstrate that there are indeed many sequences that act as *cis*-regulatory elements in 3′UTRs. Moreover, a detailed study performed on a single 3′UTR identified many discrete *cis*-regulatory elements, which had additive effects on expression [11]. Each 3′UTR having many *cis*-regulatory elements could potentially allow the cell to respond to environmental signaling by altering expression of RNA binding proteins, thus amplifying an mRNA’s repression or activation when necessary.

A growing body of evidence implies that the efficacy of a *cis*-regulatory element depends upon surrounding 3′UTR sequence context. In particular, it is clear that the efficacy of miRNA targeting is dependent on multiple local and global characteristics of the 3′UTR sequence [15, 32, 34]. Our validation experiments with endogenous 3′UTRs demonstrate that sequence context also influences the efficacy of regulatory sites we found. Because all miRNAs have a similar structure and interact with the same complement of proteins, whereas each RNA binding domain uses different structural properties to recognize their cognate motifs, the rules governing RBP accessibility are likely quite variable. Further screens and other high-throughput approaches, including CLIP-seq [35, 36], could be used to directly test the importance of sequence context by systematically examining the influence of local sequence context on *cis*-regulatory elements.

Global studies investigating alternative cleavage and polyadenylation have found that shorter 3′UTR isoforms often, although not exclusively, correlate with increased gene expression [29, 30]. One interpretation of these results is that 3′UTRs predominantly consist of repressive elements, and longer 3′UTR isoforms are more likely to accumulate increasing numbers of repressive elements, such as miRNA binding sites. It is worth noting, however, that miRNA binding sites tend to lose their efficacy in long 3′UTRs [32]. Interestingly, many of the *cis*-regulatory elements within the *HMGA2* 3′UTR, one of the few 3′UTRs for which a detailed accounting of regulatory sequences has been undertaken, act by increasing gene expression [11]. Our current work is in agreement with the *HMGA2* study; we found more 8mers that had an activating rather than repressive effect on gene expression. Importantly, activating 8mers include those that are preferentially conserved in 3′UTRs, and such conservation strongly implies that these sequences have functional roles in endogenous genes. Interestingly, 3′UTR length itself has been suggested as repressive, independent of the presence of specific regulatory elements [37]. Taken together, these results suggest that the connection between 3′UTR size and repressive activity is more intricate than previously appreciated. Nevertheless, our results clearly imply that activating elements are relatively common in mammalian 3′UTRs.

## Conclusions

We used a cell-based fluorescence screen to discover hundreds of novel post-transcriptional *cis*-regulatory elements that can alter gene regulation. Because the vast majority of these sequences are not complementary to miRNAs expressed in the cell type we used, we expect that they mediate expression by interacting with RNA binding proteins. Additionally, most of the regulatory 8mers we identified increase gene expression, reinforcing their independence from miRNA-mediated effects. The *cis*-regulatory elements we discovered are functional within endogenous 3′UTRs. Because we found hundreds of regulatory 8mers, these results suggest that each human 3′UTR is regulated by many *cis*-regulatory elements. Thus, it is clear that in many regards, our understanding of the regulatory language of 3′UTRs remains in its infancy.

## Methods

### Reporter construction

GFP was PCR amplified from pLVX-AcGFP1-N1 (Clontech) (primers listed in Additional file 17), then inserted into pEF5/FRT/V5-D-TOPO (Invitrogen) using TOPO cloning. A *Xho*I site was ablated in the vector using QuikChange Site Directed Mutagenesis (Agilent). The *PCDHB13* 3′UTR was isolated from human DNA (ATCC) and cloned into a vector containing a PGK promoter and dsRed; the PGK/dsRed/*PCDHB13* cassette was inserted into the FLP vector. The *IQGAP1* 3′UTR was isolated via PCR from human DNA (ATCC) and inserted downstream of GFP. A linker containing restriction enzyme sites for *Avr*II, *Xho*I, *Bam*HI, and *Nsi*I was inserted into the *IQGAP1* 3′UTR at a unique *Pml*I site. All 8mers were flanked by *Xho*I and *Bam*HI restriction enzyme sites and were inserted into the linker.

The *IQGAP1* 3′UTR and the polyadenylation sequence from the bovine growth hormone gene was isolated from the above construct via PCR and inserted downstream of Firefly luciferase in pMirGlo (Promega) and downstream of Renilla luciferase in pIS1 (Addgene plasmid #12179).

The resulting plasmids were validated by Sanger sequencing.

### Insert generation for 8mers

For individual 8mers, two oligonucleotides (IDT) were synthesized so that when they were annealed, they generated termini corresponding to sites digested by *Xho*I (5′ terminus) and *Bam*HI (3′ terminus) of the 8mer (Additional file 17). The resulting plasmids were validated by Sanger sequencing.

For random 8mer libraries, an oligonucleotide containing a *Xho*I site, a random 8 nucleotide sequence, a *Bam*HI site, a hairpin sequence, and a *Bam*HI site was synthesized by IDT (Additional file 17). The oligonucleotide was annealed by heating to 95 °C for 5 min in the presence of Buffer 2 (NEB) and 0.2 mM dNTPs, then cooled on ice for 3 min, thus creating a partially double stranded structure and internally primed substrate for DNA polymerase. Second strand synthesis was performed by adding 5U Klenow Fragment (3′- > 5′ exo-, NEB) and incubating for 30 min at 37 °C. This hairpin was PAGE purified on a 12 % non-denaturing gel. The hairpin was digested with *Xho*I and *Bam*HI (NEB), and digestion products were PAGE purified on a 12 % non-denaturing gel.

The oligonucleotides ordered on a microarray (DNA OligoMix, LC Sciences) consisted of *Xho*I/(N)_8_/*Bam*HI flanked by a barcode sequence and the 5′ and 3′ Illumina adapter sequences. The oligonucleotide pools were PCR amplified with Phusion polymerase (ThermoFisher Scientific), PAGE purified, digested with *Xho*I and *Bam*HI (NEB), and PAGE purified.

### Plasmid library generation

The FLP plasmid described above was digested with *Bam*HI and *Xho*I, then purified with the Wizard SV Gel and PCR Clean-Up System (Promega). 50 ng of digested plasmid and 2.3 ng of digested insert were ligated with T4 DNA Ligase (NEB) at 16 °C for 6 h, then transformed into XL-10 Gold Ultracompetent Cells (Agilent). The bacteria were transferred to liquid LB-Ampicillin, grown until saturated, and Maxi-Prepped (Promega). Plasmid libraries were validated using Illumina sequencing.

### Cell culture

Flp-In T-REx 293 cells (Life Technologies) were maintained in DMEM (Gibco) supplemented with 10 % FBS, 1 % penicillin/streptomycin, and 100 μg/mL zeocin (Invitrogen) at 37 °C with 5 % CO_2_. After stable transfection with FLP plasmids, cells were maintained in media containing 80 μg/mL hygromycin, omitting zeocin.

### Transfections

For transient transfections with miRNAs, 5x10^4^ Flp-In T-REx 293 cells were plated in 24-well plates at 24 h prior to transfection. Each well was transfected with 10 ng pIS0 (Addgene plasmid #12178), 10 ng experimental plasmid (derived from pIS1; Addgene plasmid #12179), and 25 nmol of miRNA mimic (Dharmacon) using Lipofectamine 2000 (Life Technologies). Cell were harvested 24 h later and stored at −80 °C. The sequences of the miRNA mimics were:

**Table:** 

miR-124 sense	UAAGGCACGCGGUGAAUGCCA
miR-124 anti-sense	GCAUUCACCGCGUGCCUUAAU
miR-196 sense	UAGGUAGUUUCAUGUUGUUGGG
miR-196 anti-sense	CAACAACAUGAAACUACUUAAG

For transient transfections without miRNAs that used plasmids derived from pIS1, 10^5^ Flp-In T-REx 293 cells were plated in 24-well plates at 24 h prior to transfection. Each well was transfected with 10 ng pIS0 and 15 ng experimental plasmid using Lipofectamine 2000 (Invitrogen). Cells were harvested 30 h later and stored at −80 °C. For plasmids derived from pMirGlo, 7.5x10^4^ Flp-In T-REx 293 cells were plated in 24-well plates at 24 h prior to transfection. Each well was transfected with 140 ng pUC19 and 5 ng experimental plasmid using Lipofectamine 2000 (Life Technologies). Cell were harvested 30 h later and stored at −80 °C.

For stable transfections with Flp-In plasmids, 4.5x10^6^ Flp-In T-REx 293 cells were plated per 10 cm^2^ dish in DMEM (Gibco) supplemented with 10 % FBS. Each dish was transfected 24 h later with 3 μg of library plasmids and 3.75 μg of pOG44 (which encodes the FLP recombinase; Life Technologies) using Lipofectamine 2000 (Life Technologies). Twenty-four hours post-transfection, the media was replaced with DMEM (Gibco) supplemented with 10 % FBS and 1 % penicillin/streptomycin. Selection for stably integrated cells began twenty-four hours later by replacing media with DMEM (Gibco) supplemented with 10 % FBS, 1 % penicillin/streptomycin, and 80 μg/mL hygromycin. Media changes were performed every 3–4 days until cell colonies were visible. At that point, the cells were removed from plates using trypsin, disaggregated, and mixed in 150 cm^2^ tissue-culture flasks. Cells were split every two-three days and maintained at 10–90 % confluence.

### Luciferase assays

Luciferase values for Firefly and Renilla were measured using the Dual-Luciferase Reporter Assay system (Promega) with a dual-injection luminometer (Turner Biosystems).

### Flow cytometry

Cells were sorted on a FACSAria (BD Biosciences), using a 488 nm laser and 510/21 bandpass filter for GFP, and a 532 nm laser and 575/25 bandpass filter for dsRed. Single cells were determined by their forward and side scatter profiles (Additional file 6). Of cells that were dsRed + and GFP+, the cells with either the middle 25 % or 50 % (centered on the mode of the distribution) were sorted. GFP+ sub-populations were sorted from cells with the middle 50 % of dsRed intensity. During sorting, replicates were collected into individual tubes and maintained separately. To measure fluorescence without sorting, data were acquired on a FACS LSRII instrument using DiVa software (BD Biosciences). Analysis was performed using FlowJo software (Tree Star).

### Sequencing library preparation

For each sorted sample, DNA was isolated from 2x10^7^ Flp-In T-REx 293 cells using the Blood and Cell Culture Midi Kit (Qiagen). PCR with Phusion polymerase (ThermoFisher Scientific) was then used to amplify reporter constructs that integrated at the correct position only by using a 5′ primer within the GFP gene and a 3′ primer in the Zeocin resistance gene, which is downstream of the hygromycin resistance gene at correctly integrated sites. We then used PCR to add individual barcodes and Illumina sequencing adapters to the region surrounding the variable 8-nt region for each sample; all oligonucleotides are listed in Additional file 17. We sequenced the resulting libraries on a HiSeq 2500 (Illumina), generating 50 nt reads. Each library contained 5.6x10^6^-1.6x10^7^ reads.

RNA was isolated from 10^6^ cells using Trizol (ThermoFisher Scientific). cDNA synthesis was performed with poly(dt) priming and SuperScript II reverse transcriptase (Invitrogen). To isolate 8mers in RNA, we performed PCR using Phusion polymerase (ThermoFisher Scientific) with a primer spanning the intron in EF1a, the promoter driving GFP expression. We then used PCR to add barcodes and Illumina sequencing adapters to the region surrounding the variable 8-nt region; all oligonucleotides are listed in Additional file 17. We sequenced the resulting libraries on a HiSeq 2500 (Illumina), generating 50 nt reads.

Reads from the resulting sequencing libraries were required to have Phred quality scores >20 in the barcode and variable 8mer. They were also required to have the correct sequences (upstream CATAC and downstream ATA) flanking the variable 8mer. For reads that passed these quality control filters, the number of times each 8mer was present in each library was counted, and normalized to the number of reads in the sequencing library. Details on the barcodes and sequences of the resulting reads are provided in Additional file 18.

### Metrics for scoring 8mers

An 8mer’s enrichment in sorted GFP+ sub-populations was found by calculating its normalized read count (reads per million, RPM) in each sub-population, then dividing each of the resulting RPM values by the 8mer’s RPM value in the dsRed middle-50 % cells.

An 8mer’s expression score was calculated by scaling its RPM value from each sorting bin to its bin value (−2 for 0–10 %, −1 for 20–30 %, 0 for 40–60 %, 1 for 70–80 %, 2 for 90–100 %). The resulting scaled-RPMs were then summed across the five bins, and an average score for each 8mer calculated by dividing that sum by the total RPMs from the five bins.

### Comparative analysis of 8mer sequences

Aligned 3′UTR sequences were extracted from the UCSC genome browser [38, 39], and the number of conserved instances of each 8mer calculated. Sites were considered conserved if the sequence was identical in the human, mouse, rat, and dog genomes. For each 8mer sequence, the number of conserved counts was judged against the average for a set of control shuffled 8mers [40].

### UTR cloning

Fragments of 3′UTRs that were 400–600 nucleotides long and centered on the 8mer were amplified from human DNA (ATCC) using Phusion (ThermoFisher Scientific) with oligonucleotides that had a *Sal*I site on the 5′ primer and *Not*I site on the 3′ primer. Inserts were digested with *Not*I-HF and *Sal*I-HF (NEB), then 25 ng of digested insert was ligated to 25 ng of pMirGlo (Promega) that had been digested with *Not*I-HF and *Sal*I-HF. Mutations were generated in the 8mer using QuikChange Site Directed Mutagenesis (Agilent). All plasmids were sequence verified. Sequences of the UTRs that were cloned are available in Additional file 19.

### Decay experiments

For each time point, 5x10^5^ cells were plated per well of a 6-well plate, and twenty-four hours later, the media was replaced with media supplemented with 2.5 μg/mL actinomycin D (Life Technologies). At each time point, media was removed and cells were placed in Trizol (ThermoFisher Scientific), then stored at −80 °C prior to RNA isolation. cDNA synthesis was performed with poly(dT) oligonucleotide (IDT) and RevertAid Reverse Transcriptase (ThermoFisher Scientific). qPCR reactions were performed using Taq polymerase and SYBR Green (Life Technologies) as the detection agent, using GAPDH as a normalization gene. Each qPCR reaction was done in triplicate, and performed on at least two biological replicate samples. Primer sequences used for quantitative PCR are described in Additional file 17.

### Enrichment statistics

For each category of tested 8mers, we found the number of genes that were located in both the category and the activating or repressive set (b), the total number of genes present in that category (*n*), the number of genes defined as activating or repressive (B), and the total number of 8mers screened (*N*). Enrichment was calculated as (b/n)/(B/N). Two-sided Fisher exact tests were used to determine significance.

### Small RNA sequencing

RNA was isolated using Trizol (Life Technologies), and 1 μg of total RNA was used to generate small RNA libraries using the TruSeq Small RNA Prep Kit (Illumina). miRNA expression was found with MirDeep2 [41] and Bowtie [42] (hg19), using miRBase version 21 [43]. Read counts from miRNA families (miRNAs with the same seed sequence) were combined.

## Availability of supporting data

The data sets supporting the results of this article are available in the GEO repository [44]: [GEO:GSE75161]. Plasmids are available upon request.

## Additional file**s**

Additional file 1: Details of the experimental design. (PDF 375 kb) Additional file 2: Figure showing pilot screen data. (PDF 434 kb) Additional file 3: Read counts for each 8mer from the pilot screen. (XLS 1369 kb) Additional file 4: 8mer categories tested in the screen. (XLS 28 kb) Additional file 5: Site-specific integration details. (PDF 115 kb) Additional file 6: FACS gating strategy. (PDF 387 kb) Additional file 7: Figure showing reproducibility of fluorescence for sorting replicates. (PDF 274 kb) Additional file 8: Figure showing reproducibility of sequencing data for sorting replicates. (PDF 375 kb) Additional file 9: Read counts for each 8mer in the main screen. (XLS 868 kb) Additional file 10: Figure showing each 8mer’s score and enrichment values in GFP sub-populations. (PDF 247 kb) Additional file 11: Validation of 8mers found in the main screen. (PDF 280 kb) Additional file 12: miRNA expression in HEK293-FLP cells and expression scores of miRNA targets. (XLS 140 kb) Additional file 13: RBP motifs that were tested in the main screen. (XLS 25 kb) Additional file 14: Figure showing the robustness of our enrichment analysis. (PDF 63 kb) Additional file 15: Figure of reverse complement pairs. (PDF 128 kb) Additional file 16: mRNA half-life data. (PDF 293 kb) Additional file 17: Primers used in this study. (XLS 42 kb) Additional file 18: Barcoded sequencing reads produced. (XLS 26 kb) Additional file 19: Native 3′UTR sequences used in Fig. 6. (PDF 130 kb)

## References

[1] Kuersten S, Goodwin EB (2003). The power of the 3′ UTR: translational control and development. Nat Rev Genet.

[2] Matoulkova E, Michalova E, Vojtesek B, Hrstka R (2012). The role of the 3’ untranslated region in post-transcriptional regulation of protein expression in mammalian cells. RNA Biol.

[3] Zhao W, Blagev D, Pollack JL, Erle DJ (2011). Toward a systematic understanding of mRNA 3′ untranslated regions. Proc Am Thorac Soc.

[4] Siepel A, Bejerano G, Pedersen JS, Hinrichs AS, Hou M, Rosenbloom K (2005). Evolutionarily conserved elements in vertebrate, insect, worm, and yeast genomes. Genome Res.

[5] Xie X, Lu J, Kulbokas EJ, Golub TR, Mootha V, Lindblad-Toh K (2005). Systematic discovery of regulatory motifs in human promoters and 3′ UTRs by comparison of several mammals. Nature.

[6] Friedman RC, Farh KK-H, Burge CB, Bartel DP (2009). Most mammalian mRNAs are conserved targets of microRNAs. Genome Res.

[7] Bartel DP (2009). MicroRNAs: target recognition and regulatory functions. Cell.

[8] Baltz AG, Munschauer M, Schwanhäusser B, Vasile A, Murakawa Y, Schueler M (2012). The mRNA-bound proteome and its global occupancy profile on protein-coding transcripts. Mol Cell.

[9] Castello A, Fischer B, Eichelbaum K, Horos R, Beckmann BM, Strein C (2012). Insights into RNA biology from an atlas of mammalian mRNA-binding proteins. Cell.

[10] Kwon SC, Yi H, Eichelbaum K, Föhr S, Fischer B, You KT (2013). The RNA-binding protein repertoire of embryonic stem cells. Nat Struct Mol Biol.

[11] Kristjánsdóttir K, Fogarty EA, Grimson A (2015). Systematic analysis of the Hmga2 3′ UTR identifies many independent regulatory sequences and a novel interaction between distal sites. RNA.

[12] Zhao W, Pollack JL, Blagev DP, Zaitlen N, McManus MT, Erle DJ (2014). Massively parallel functional annotation of 3′ untranslated regions. Nat Biotechnol.

[13] Khaziapoul S, Pearson MJ, Pryme IF, Stern B, Hesketh JE (2012). CUG binding protein 1 binds to a specific region within the human albumin 3′ untranslated region. Biochem Biophys Res Commun.

[14] Wirsing A, Senkel S, Klein-Hitpass L, Ryffel GU (2011). A systematic analysis of the 3′UTR of HNF4A mRNA reveals an interplay of regulatory elements including miRNA target sites. PLoS One.

[15] Agarwal V, Bell GW, Nam J-W, Bartel DP (2015). Predicting effective microRNA target sites in mammalian mRNAs. eLife.

[16] Auweter SD, Oberstrass FC, Allain FH-T (2006). Sequence-specific binding of single-stranded RNA: is there a code for recognition?. Nucleic Acids Res.

[17] Gerstberger S, Hafner M, Tuschl T (2014). A census of human RNA-binding proteins. Nat Rev Genet.

[18] Masliah G, Barraud P, Allain FH-T (2013). RNA recognition by double-stranded RNA binding domains: a matter of shape and sequence. Cell Mol Life Sci CMLS.

[19] Mitchell SF, Parker R (2014). Principles and properties of eukaryotic mRNPs. Mol Cell.

[20] Lambert N, Robertson A, Jangi M, McGeary S, Sharp PA, Burge CB (2014). RNA bind-n-Seq: quantitative assessment of the sequence and structural binding specificity of RNA binding proteins. Mol Cell.

[21] Ray D, Kazan H, Cook KB, Weirauch MT, Najafabadi HS, Li X (2013). A compendium of RNA-binding motifs for decoding gene regulation. Nature.

[22] Wang Z, Rolish ME, Yeo G, Tung V, Mawson M, Burge CB (2004). Systematic identification and analysis of exonic splicing silencers. Cell.

[23] Dean KM, Grayhack EJ (2012). RNA-ID, a highly sensitive and robust method to identify cis-regulatory sequences using superfolder GFP and a fluorescence-based assay. RNA.

[24] Arnold CD, Gerlach D, Stelzer C, Boryń ŁM, Rath M, Stark A (2013). Genome-wide quantitative enhancer activity maps identified by STARR-seq. Science.

[25] Oikonomou P, Goodarzi H, Tavazoie S (2014). Systematic identification of regulatory elements in conserved 3′ UTRs of human transcripts. Cell Rep.

[26] Naftelberg S, Schor IE, Ast G, Kornblihtt AR (2015). Regulation of alternative splicing through coupling with transcription and chromatin structure. Annu Rev Biochem.

[27] Nott A, Hir HL, Moore MJ (2004). Splicing enhances translation in mammalian cells: an additional function of the exon junction complex. Genes Dev.

[28] Moore MJ, Proudfoot NJ (2009). Pre-mRNA processing reaches back toTranscription and ahead to translation. Cell.

[29] Mayr C, Bartel DP (2009). Widespread shortening of 3′UTRs by alternative cleavage and polyadenylation activates oncogenes in cancer cells. Cell.

[30] Sandberg R, Neilson JR, Sarma A, Sharp PA, Burge CB (2008). Proliferating cells express mRNAs with shortened 3′ untranslated regions and fewer microRNA target sites. Science.

[31] Fabian MR, Sonenberg N (2012). The mechanics of miRNA-mediated gene silencing: a look under the hood of miRISC. Nat Struct Mol Biol.

[32] Grimson A, Farh KK-H, Johnston WK, Garrett-Engele P, Lim LP, Bartel DP (2007). MicroRNA targeting specificity in mammals: determinants beyond seed pairing. Mol Cell.

[33] Lebedeva S, Jens M, Theil K, Schwanhäusser B, Selbach M, Landthaler M (2011). Transcriptome-wide analysis of regulatory interactions of the RNA-binding protein HuR. Mol Cell.

[34] Garcia DM, Baek D, Shin C, Bell GW, Grimson A, Bartel DP (2011). Weak seed-pairing stability and high target-site abundance decrease the proficiency of lsy-6 and other microRNAs. Nat Struct Mol Biol.

[35] Hafner M, Landthaler M, Burger L, Khorshid M, Hausser J, Berninger P (2010). Transcriptome-wide identification of RNA-binding protein and MicroRNA target sites by PAR-CLIP. Cell.

[36] Licatalosi DD, Mele A, Fak JJ, Ule J, Kayikci M, Chi SW (2008). HITS-CLIP yields genome-wide insights into brain alternative RNA processing. Nature.

[37] Hogg JR, Goff SP (2010). Upf1 senses 3′UTR length to potentiate mRNA decay. Cell.

[38] Karolchik D, Hinrichs AS, Furey TS, Roskin KM, Sugnet CW, Haussler D (2004). The UCSC table browser data retrieval tool. Nucleic Acids Res.

[39] Kent WJ, Sugnet CW, Furey TS, Roskin KM, Pringle TH, Zahler AM (2002). The human genome browser at UCSC. Genome Res.

[40] Lewis BP, Burge CB, Bartel DP (2005). Conserved seed pairing, often flanked by adenosines, indicates that thousands of human genes are MicroRNA targets. Cell.

[41] Friedländer MR, Mackowiak SD, Li N, Chen W, Rajewsky N (2012). miRDeep2 accurately identifies known and hundreds of novel microRNA genes in seven animal clades. Nucleic Acids Res.

[42] Langmead B, Salzberg SL (2012). Fast gapped-read alignment with Bowtie 2. Nat Methods.

[43] Kozomara A, Griffiths-Jones S (2014). miRBase: annotating high confidence microRNAs using deep sequencing data. Nucleic Acids Res.

[44] Edgar R, Domrachev M, Lash AE (2002). Gene expression omnibus: NCBI gene expression and hybridization array data repository. Nucleic Acids Res.

