# Supplementary material for: High-throughput discovery of post-transcriptional cis-regulatory elements
Source: BMC Genomics. 2016 Mar 3;17:177. doi: 10.1186/s12864-016-2479-7 (PMC4778349; doi:10.1186/s12864-016-2479-7)
Supplement: Additional file 1: — Details of the experimental design. (PDF 375 kb) [file 12864_2016_2479_MOESM1_ESM.pdf]

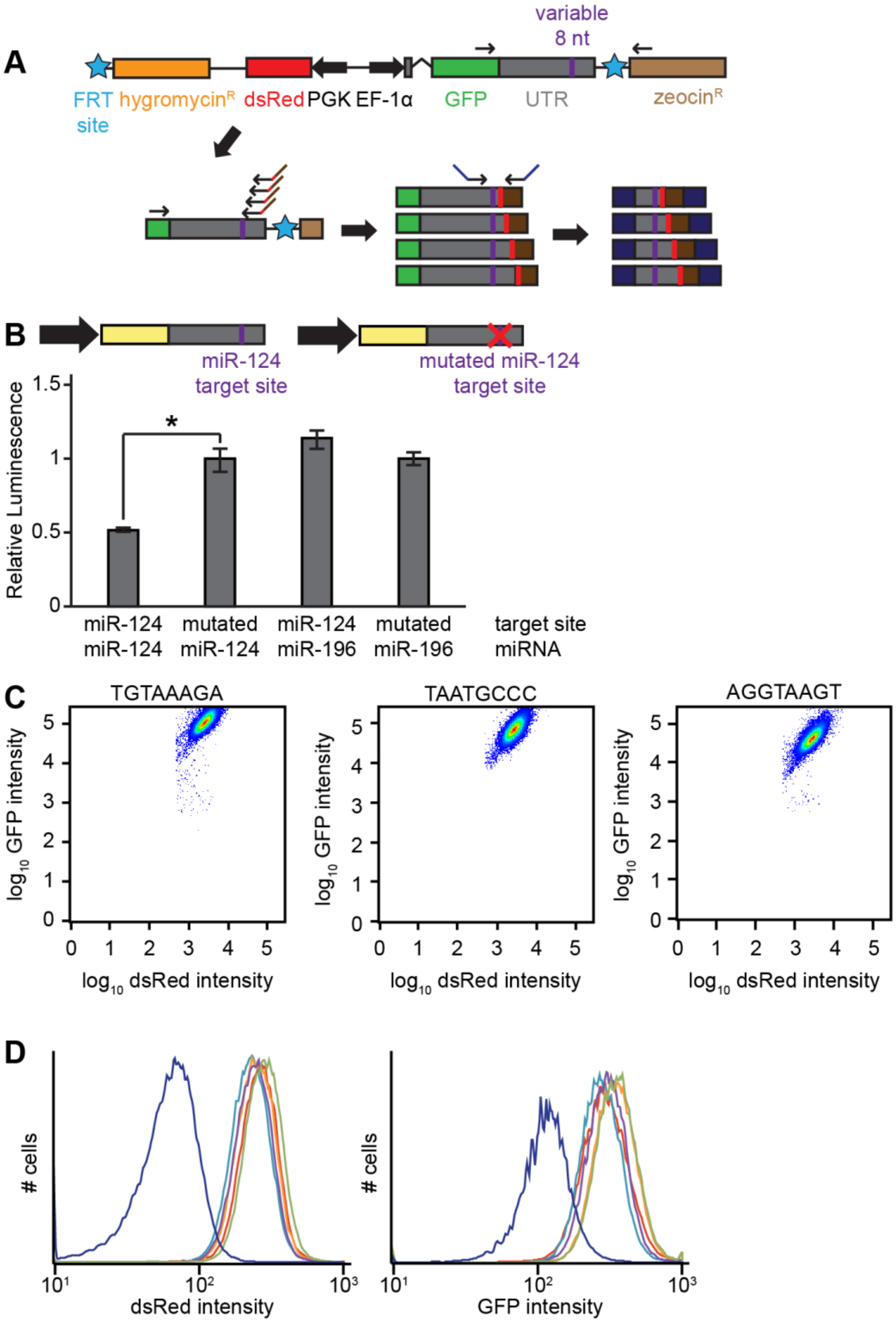

Additional file 1. Experimental design details. (A) Sequencing strategy. To ensure that we only analyzed cells in which the reporter gene had integrated into the correct region of the genome, we used a reverse primer specific to the zeocin resistance gene, which is downstream of the FRT-site in 293-FLP cells. The forward primer is located within the GFP sequence. Following this initial round of PCR, we performed additional rounds of PCT with the same

forward primer, within GFP, and reverse primers that included the sequence corresponding to the Illumina sequencing primer (shown in brown) together with a barcode for each library. These barcoded primers were designed so that Illumina sequencing of the 3′UTR began at different registers, thus increasing the complexity of the sequencing library; the barcodes (shown in red) each differed from one another at two variable nucleotide positions. Illumina sequencing adapters (shown in blue) are added in the final rounds of amplification. (B) The human *IQGAP1* 3′UTR can be regulated by exogenous 8mers. An intact or mutated miR-124 target site was inserted into a luciferase-*IQGAP1* 3′UTR, using the same *IQGAP1* insertion position used in Figure 1. The luciferase activity of constructs containing the intact miR-124 target site was normalized to the construct with a disrupted target site, in the presence of miR-124 or miR-196. Reporter data were plotted as the geometric mean of relative luminescence (y-axis) of reporter constructs normalized to those with disrupted sites; error bars indicate 68% of the data. Significance was determined by a two-sided Wilcoxon rank sum test, n=6, *p<0.005. (C) A second fluorophore controls for transcriptional noise at the reporter locus. The FLP vector shown in Figure 1 had three specific 8mers inserted into the *IQGAP1* 3′UTR, and those vectors were integrated into the genome of HEK-293 FLP cells. Cells with unusually high or low GFP expression also had unusually high or low dsRed expression. (D) HEK-293 FLP cells with one integrated 8mer were clonalized. The dsRed and GFP fluorescence intensities were measured for each clone; clones are depicted with the same color in the two plots. The single clone (in blue) with anomalous GFP reporter activity exhibited a corresponding decrease in deRed activity.
