# Supplementary material for: High-throughput discovery of post-transcriptional cis-regulatory elements
Source: BMC Genomics. 2016 Mar 3;17:177. doi: 10.1186/s12864-016-2479-7 (PMC4778349; doi:10.1186/s12864-016-2479-7)
Supplement: Additional file 2: — Figure showing pilot screen data. (PDF 434 kb) [file 12864_2016_2479_MOESM2_ESM.pdf]

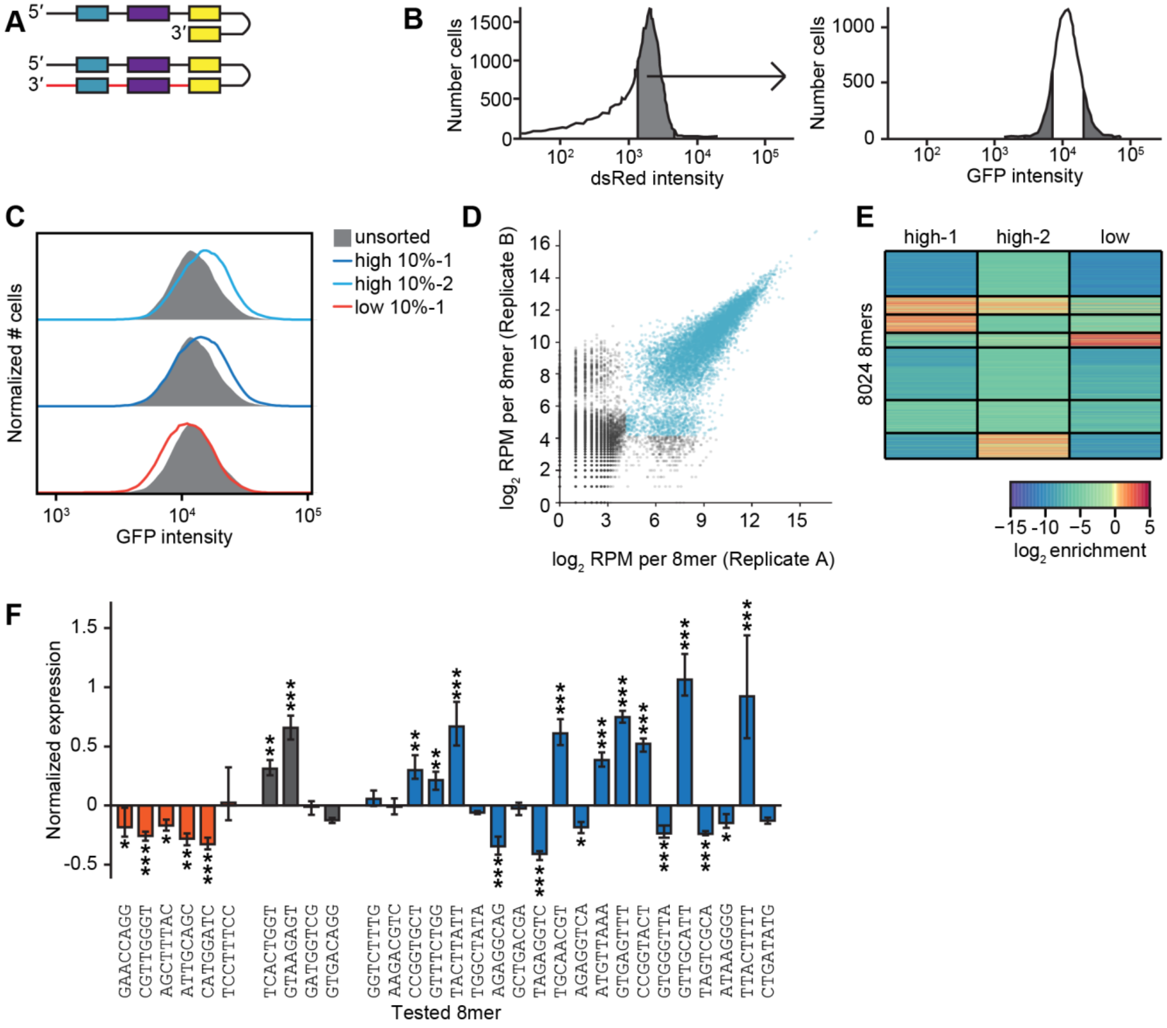

Additional file 2. Measuring the regulatory potential of thousands of short sequence elements. (A) Insert generation. An oligonucleotide containing a *Xho*I recognition site (shown in blue), two *Bam*HI recognition sites (yellow), and a random 8mer (purple) was annealed, then the second strand of the 8mer was generated by Klenow. (B) Sorting strategy. Random 8mers were inserted into the *IQGAP1* 3′UTR-GFP-FLP plasmid, and the resulting plasmid library was integrated into HEK293-TRex-FLP cells. Fluorescence activated cell sorting (FACS) was used to isolate cells that had a narrow window of GFP and dsRed transcription while also having differential post-transcriptional regulation of GFP. Transcription was controlled by selecting cells

with the middle 50% of dsRed expression (shown in gray). Of those cells, the top and bottom 10% of GFP-expressing cells were sorted (shown in gray). (C) Sorted cells had heritable differences in GFP expression. Sorted cells were maintained for three weeks, then their fluorescence was measured via FACS, and their GFP intensities overlaid upon the GFP intensities for unsorted cells (in grey). The number of cells is normalized to the mode of GFP intensity. (D) 8mers were only included in the analysis if they were robustly present in two technical replicates of the sequencing data generated from the unsorted cells. The abundance of each 8mer was plotted for two of the sequencing technical replicates. There were 8,024 sequences (blue dots in the plot) robustly detected, as defined by having an abundance of at least 16 reads in both replicates. (E) Motifs are enriched in sorted populations. The relative enrichment values for each of the 8,024 well-represented 8mers were found by dividing their normalized read counts (reads per million, RPM) in each sorted population by their RPM value in unsorted cells. They were ordered by unsupervised clustering. (F) The pilot screen had a 50% validation rate. Candidate motifs were chosen from the screen based on enrichment in sorted populations. Each candidate was inserted into the *IQGAP1* 3′UTR-luciferase construct, and the luciferase activity of these reporter constructs was normalized to the UAAUGCCC element. Data are plotted as relative luminescence (y-axis) of reporters, and are represented as the geometric mean and error bars indicate 68% of the, n=9-18; significance was determined by a two-sided Wilcoxon rank sum test, *$p<0.05$, **$p<0.005$, ***$p<0.0005$. Orange motifs were predicted to downregulate expression, gray motifs were expected to have no effect, and blue motifs were expected to upregulate expression.
