# Supplementary material for: High-throughput discovery of post-transcriptional cis-regulatory elements
Source: BMC Genomics. 2016 Mar 3;17:177. doi: 10.1186/s12864-016-2479-7 (PMC4778349; doi:10.1186/s12864-016-2479-7)
Supplement: Additional file 5: — Site-specific integration details. (PDF 115 kb) [file 12864_2016_2479_MOESM5_ESM.pdf]

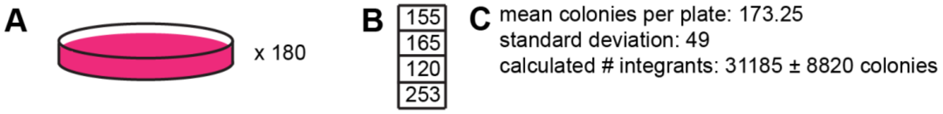

Additional file 5. Estimating number of integration events. (A) We performed the FLP/FRT transfection on forty-five 10cm^2 plates, then diluted the cells from each plate to four plates, resulting in a total of one hundred and eighty plates. (B) Counts of integration events. After selecting with hygromycin for 10-11 days, we counted the number of cell colonies, indicating individual integration events, on four plates. (C) Calculating total number integration events based on counts in B.
