# Supplementary material for: High-throughput discovery of post-transcriptional cis-regulatory elements
Source: BMC Genomics. 2016 Mar 3;17:177. doi: 10.1186/s12864-016-2479-7 (PMC4778349; doi:10.1186/s12864-016-2479-7)
Supplement: Additional file 6: — FACS gating strategy. (PDF 387 kb) [file 12864_2016_2479_MOESM6_ESM.pdf]

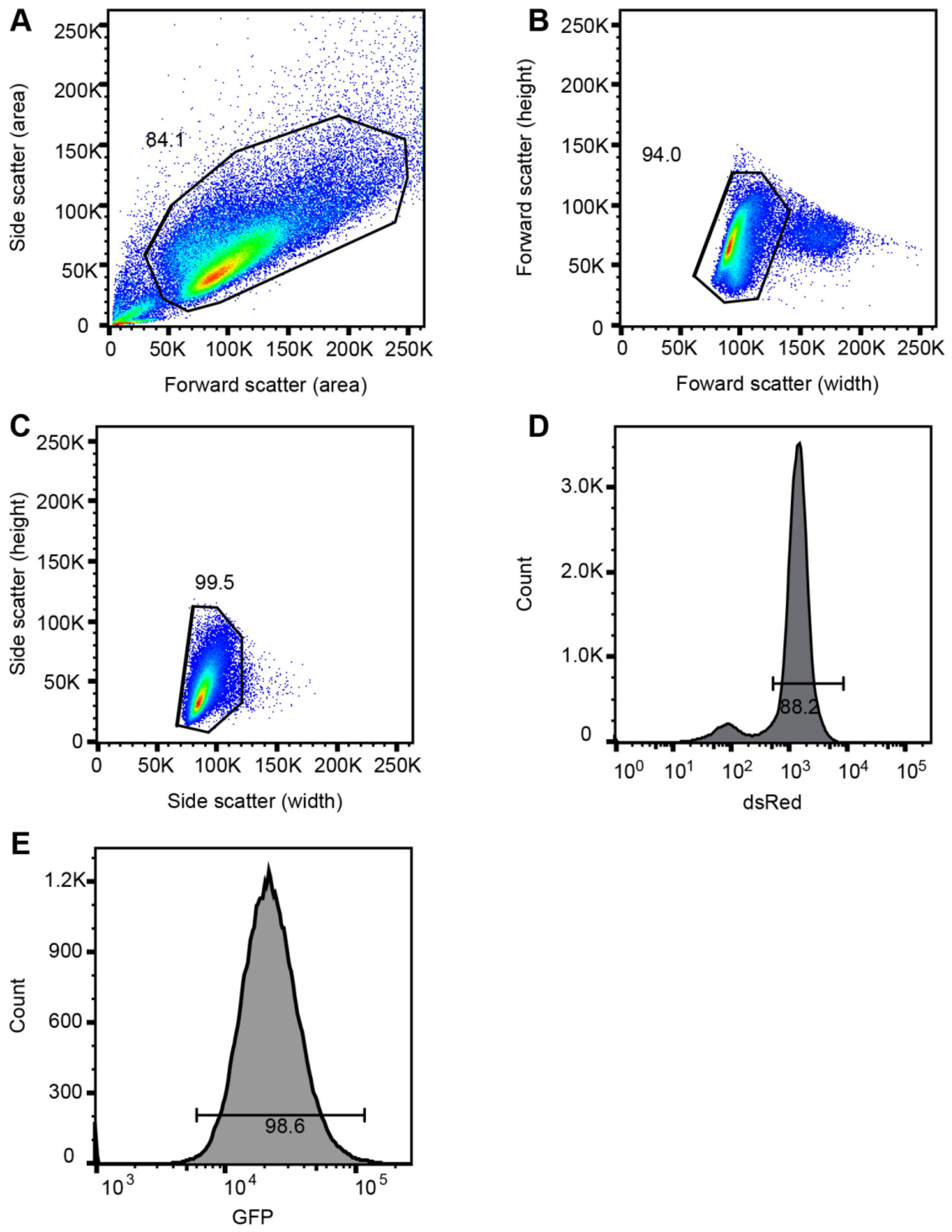

Additional file 6. Gating strategy for sorting. The gates used to select single cells that were GFP+ and dsRed+ are shown; the number in each plot signifies the percentage of cells present within the selected gates. (A) The forward scatter and side scatter were used to define the population of cells. (B) Using the cells from A, single cells were defined based on the height and width of the forward scatter. (C) Side scatter width and height were used to define cells with a

narrow range of granularity on the cells gated in B. (D) The gates used to select cells defined as dsRed+. (E) The gates used to select cells defined as GFP+.
