# Supplementary material for: High-throughput discovery of post-transcriptional cis-regulatory elements
Source: BMC Genomics. 2016 Mar 3;17:177. doi: 10.1186/s12864-016-2479-7 (PMC4778349; doi:10.1186/s12864-016-2479-7)
Supplement: Additional file 7: — Figure showing reproducibility of fluorescence for sorting replicates. (PDF 274 kb) [file 12864_2016_2479_MOESM7_ESM.pdf]

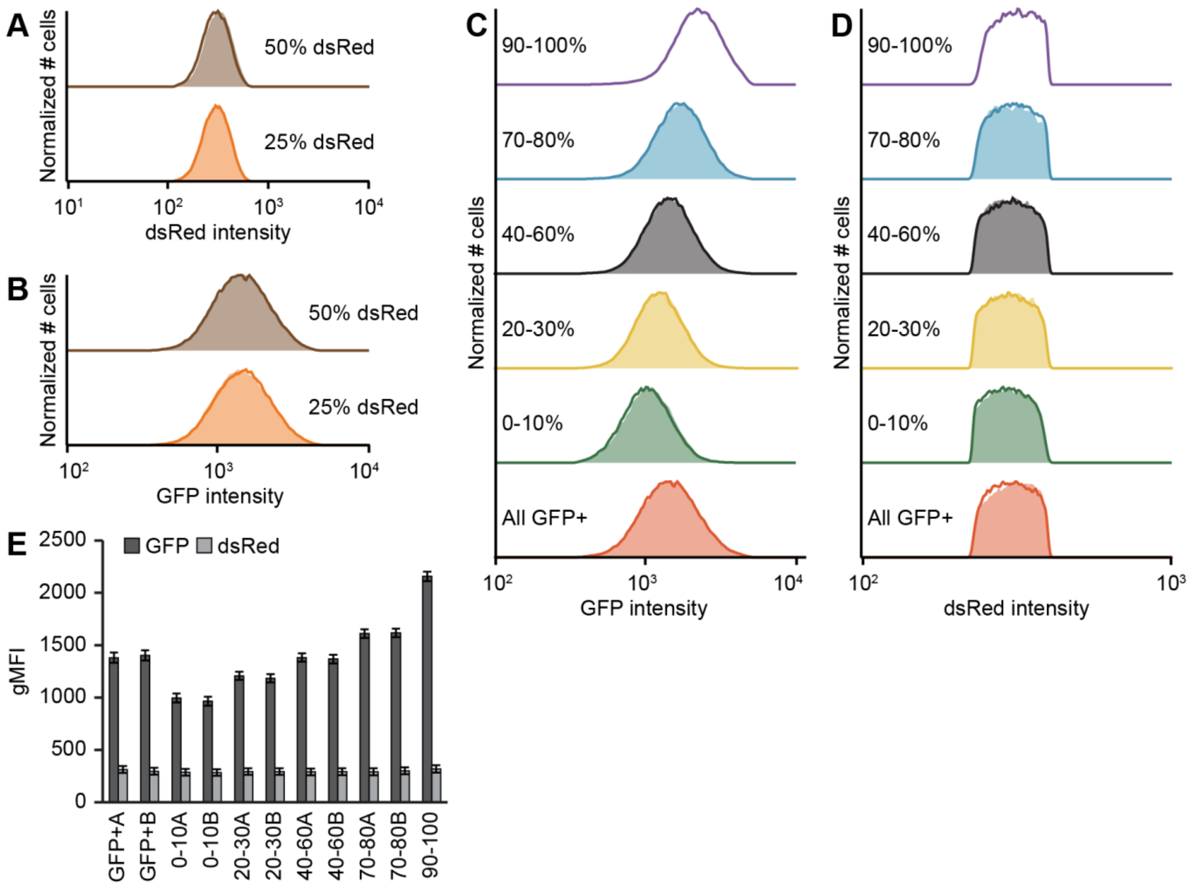

Additional file 7. Replicate sorted cells had similar fluorophore intensity distributions. Fluorescence of sorted cells was measured on an Aria LS II three weeks post-sorting. In all plots, the shaded histogram represents one sorting replicate, and the outlined histogram represents a second sorting replicate; only one replicate was recovered for the 90-100% GFP+ cells, all other GFP+ sub-populations were represented by two replicates. (A) GFP+ cells were sorted based on different dsRed intensity cutoffs (middle 50% [upper] and middle 25% [lower]). Post-sorting, the replicates have similar dsRed intensities, as do the two sorted populations. (B) GFP distributions from GFP+ cells sorted based on different dsRed intensity cutoffs are similar between replicates and between different dsRed cut-offs. (C) GFP distributions from the sorting replicates for GFP sub-populations are highly concordant. (D) dsRed distributions from all samples are similar to each other. (E) The geometric mean fluorescence intensity ± the

coefficient of variation are plotted for GFP and dsRed intensity for each sorted population using a dsRed 50% cutoff.
