# Supplementary material for: High-throughput discovery of post-transcriptional cis-regulatory elements
Source: BMC Genomics. 2016 Mar 3;17:177. doi: 10.1186/s12864-016-2479-7 (PMC4778349; doi:10.1186/s12864-016-2479-7)
Supplement: Additional file 8: — Figure showing reproducibility of sequencing data for sorting replicates. (PDF 375 kb) [file 12864_2016_2479_MOESM8_ESM.pdf]

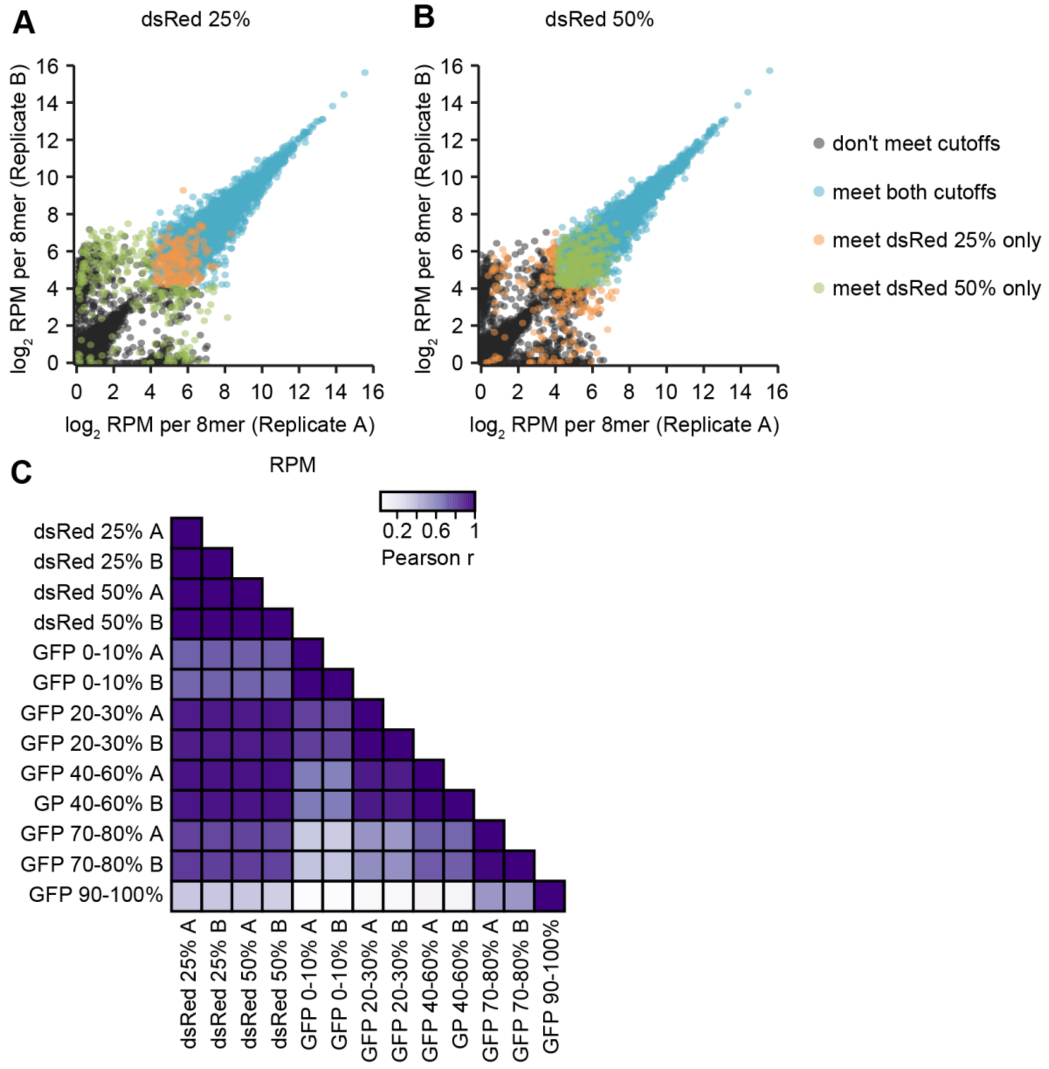

Additional file 8. Sorting replicates have well-correlated sequencing data. (A and B) 8mers were only included in the analysis if they were robustly present (>16 reads per million) in GFP+ cells isolated from both the middle 25% and 50% of the dsRed popoulation. The abundance of each 8mer was normalized by the read depth in each library (reads per million, RPM). The RPM values for each 8mer were plotted for two sorting replicates collected from dsRed 25% cells (A) and dsRed 50% cells (B). There were 2,005 robustly present sequences (blue dots in the plot), as defined by having an abundance of at least at least 2^4 RPM in both replicates of both dsRed cutoff libraries. (C) Sorting replicates have highly correlated abundances of 8mers. For each

sequencing library, the pair-wise correlations of normalized abundances each 8mer were calculated.
