# Supplementary material for: High-throughput discovery of post-transcriptional cis-regulatory elements
Source: BMC Genomics. 2016 Mar 3;17:177. doi: 10.1186/s12864-016-2479-7 (PMC4778349; doi:10.1186/s12864-016-2479-7)
Supplement: Additional file 10: — Figure showing each 8mer’s score and enrichment values in GFP sub-populations. (PDF 247 kb) [file 12864_2016_2479_MOESM10_ESM.pdf]

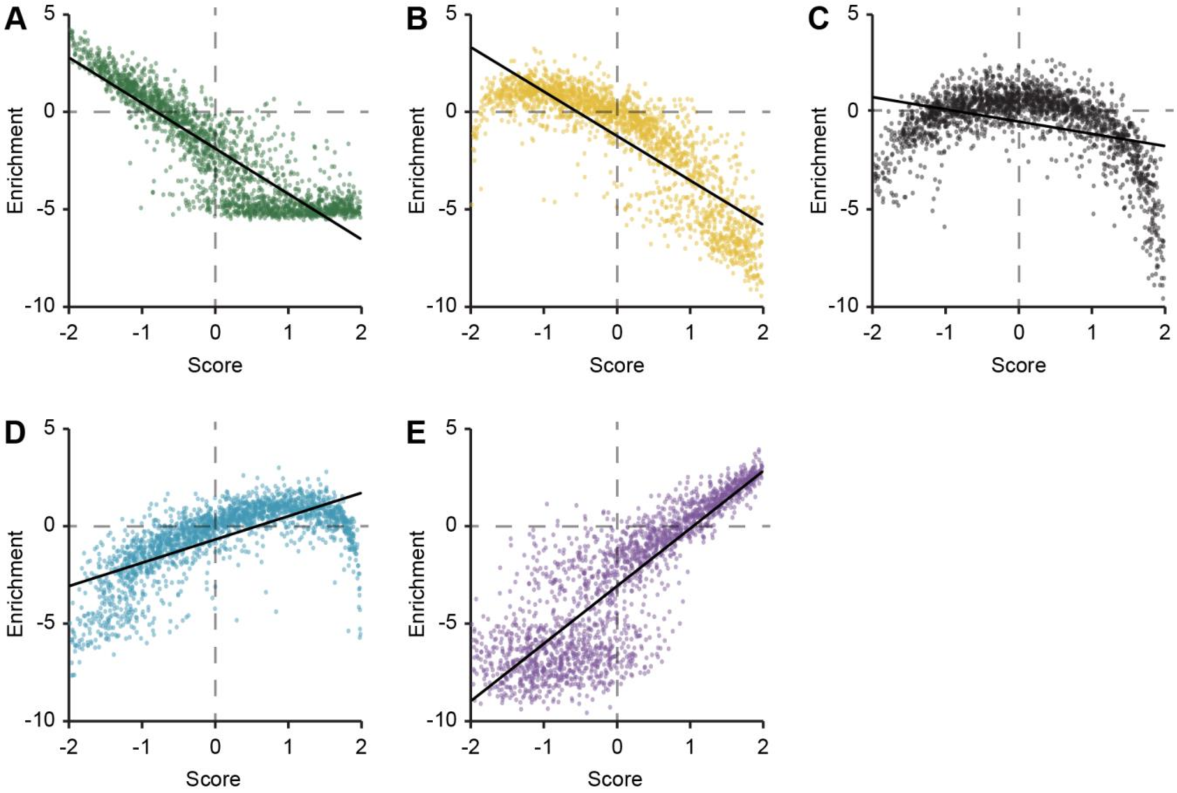

Additional file 10. Enrichment and scores for each 8mer. To examine the relation between the score metric and enrichment in each sorted GFP sub-population, we plotted each 8mer's score (x-axis) and enrichment (y-axis) for each GFP sub-population. (A) 0-10% (lowest GFP bin). Pearson r=-0.887, $p<10^{-5}$. (B) 20-30%. Pearson r=-0.831, $p<10^{-5}$. (C) 40-60%. Pearson r=-.0362, $p<10^{-5}$. (D) 70-80%. Pearson r=0.679, $p<10^{-5}$. (E) 90-100% (highest GFP bin). Pearson r=0.848, $p<10^{-5}$.
