# Supplementary material for: High-throughput discovery of post-transcriptional cis-regulatory elements
Source: BMC Genomics. 2016 Mar 3;17:177. doi: 10.1186/s12864-016-2479-7 (PMC4778349; doi:10.1186/s12864-016-2479-7)
Supplement: Additional file 11: — Validation of 8mers found in the main screen. (PDF 280 kb) [file 12864_2016_2479_MOESM11_ESM.pdf]

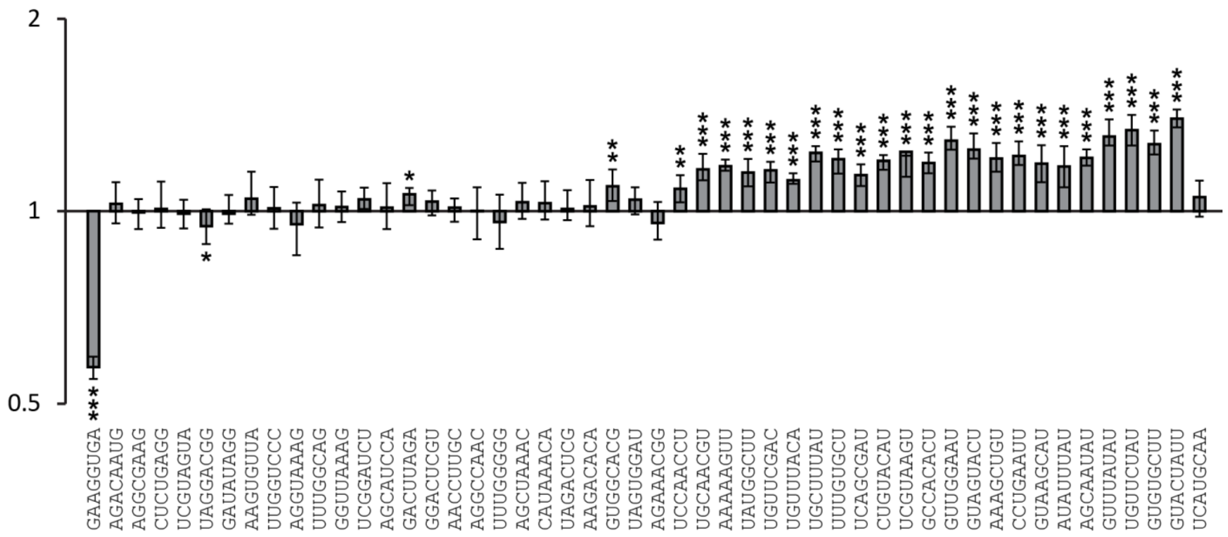

Additional file 11. Candidate elements recapitulate their predicted behavior. Each candidate was inserted into the *IQGAP1* 3′UTR-luciferase construct, and the luciferase activity of these reporter constructs were normalized to the geometric means of expression controls UUCCGUUA and UAAUGCCC. Candidate 8mers are ordered by expression score (low scores on the left, high scores on the right). Data are plotted as relative luminescence (y-axis) of reporters, and are represented as the geometric mean ± 33% of the spread of the data, n=6-18; significance was determined by a two-sided Wilcoxon rank sum test, *p<0.05, **p<0.005, ***p<0.0005.
