# Supplementary material for: High-throughput discovery of post-transcriptional cis-regulatory elements
Source: BMC Genomics. 2016 Mar 3;17:177. doi: 10.1186/s12864-016-2479-7 (PMC4778349; doi:10.1186/s12864-016-2479-7)
Supplement: Additional file 14: — Figure showing the robustness of our enrichment analysis. (PDF 63 kb) [file 12864_2016_2479_MOESM14_ESM.pdf]

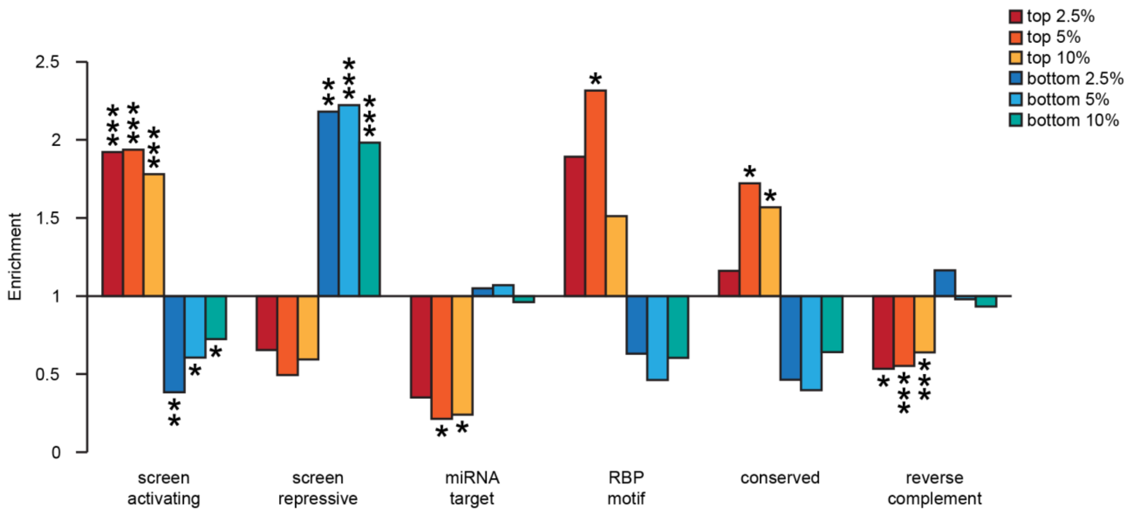


**Additional file 14. Cutoffs for activating and repressive elements are robust.** Different percentages of 8mers were considered activating or repressive, based on their expression score. Of the 2,005 8mers, 51 met the 2.5% cutoff (scores >1.84 and <-1.670), 101 met the 5% cutoff (scores >1.75 and <-1.52), and 201 met the 10% cutoff (scores >1.55 and <-1.27). For each cutoff, the enrichment of the different categories of 8mers was found. Significance was assessed by two-sided Fisher exact tests; *p<0.05, **p<0.005, ***p<0.0005.
