# Supplementary material for: High-throughput discovery of post-transcriptional cis-regulatory elements
Source: BMC Genomics. 2016 Mar 3;17:177. doi: 10.1186/s12864-016-2479-7 (PMC4778349; doi:10.1186/s12864-016-2479-7)
Supplement: Additional file 15: — Figure of reverse complement pairs. (PDF 128 kb) [file 12864_2016_2479_MOESM15_ESM.pdf]

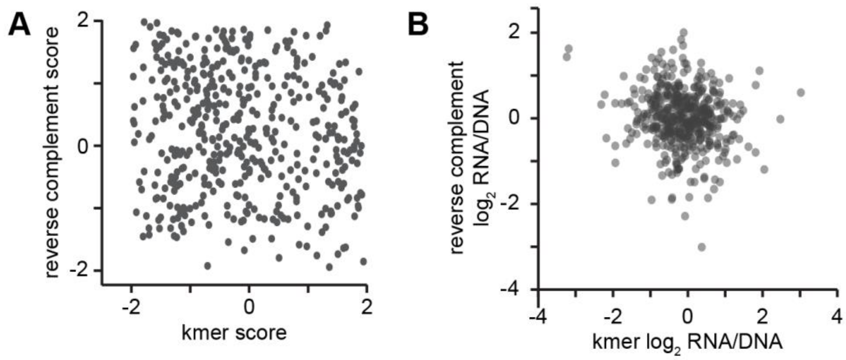


**Additional file 15. Reverse complementary pairs of sequences have poor correlations in their predicted regulatory impacts.** Pairs of 8-nt reverse complements that were robustly sequenced were identified. There were 466 pairs, 460 of which are not palindromic. (A) Expression scores for the 460 pairs of reverse complements were found. Pearson r = -0.13182, $p<0.005$. (B) RNA/DNA ratios for the 460 pairs of reverse complements were found. Pearson r = -0.14623, $p<0.005$.
