# Supplementary material for: High-throughput discovery of post-transcriptional cis-regulatory elements
Source: BMC Genomics. 2016 Mar 3;17:177. doi: 10.1186/s12864-016-2479-7 (PMC4778349; doi:10.1186/s12864-016-2479-7)
Supplement: Additional file 16: — mRNA half-life data. (PDF 293 kb) [file 12864_2016_2479_MOESM16_ESM.pdf]

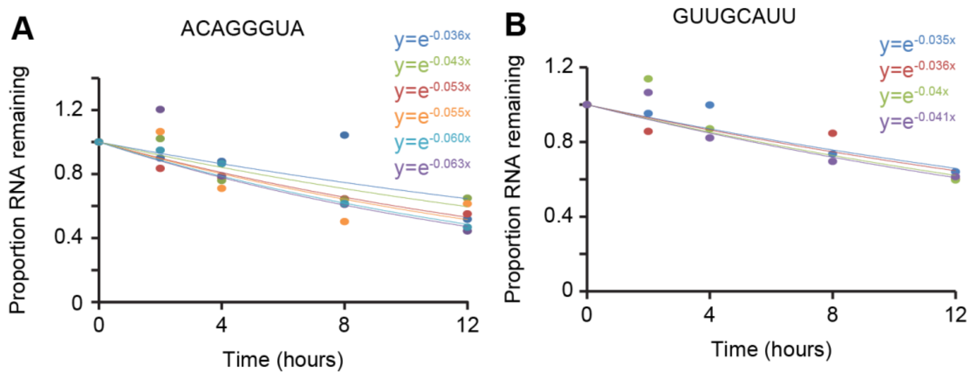

C

8mer	Rate constant (proportion RNA/hr)	Half-life (hrs)	Pearson correlation	RNA/DNA value	# replicates
AGGUAAGU	-0.0663	10.45	-0.812	-0.947	5
ACAGGGUA	-0.0517	13.41	-0.865	-0.553	6
UGUACAUA	-0.0678	10.22	-0.737	0.392	4
UGUAAAGA	-0.0425	16.32	-0.862	0.447	4
GUUGCAUU	-0.0379	18.28	-0.9278	0.796	4

Additional file 16. Half-life calculations. (A and B) Data for two individual integrated 8mers in our GFP reporter system were assayed, ACAGGGUA (A) and GUUGCAUU (B). Transcription was arrested, and cells were collected at 0, 2, 4, 8, and 12 hours, then the relative amount of reporter mRNA remaining was found. Each replicate's rate constant was found by fitting a best-fit exponential line to the data. (C) Half-life values for five 8mers, each integrated in our GFP reporter system. Rate constants and Pearson correlation values were found by combining all replicate data collected for each 8mer. Half-life was calculated as ln(2)/-rate constant.
