# Supplementary material for: High-throughput discovery of post-transcriptional cis-regulatory elements
Source: BMC Genomics. 2016 Mar 3;17:177. doi: 10.1186/s12864-016-2479-7 (PMC4778349; doi:10.1186/s12864-016-2479-7)
Supplement: Additional file 19: — Native 3′UTR sequences used in Fig. 6. (PDF 130 kb) [file 12864_2016_2479_MOESM19_ESM.pdf]

Additional file 19. Sequences of UTRs assayed in Figure 6.

Reporter plasmids were constructed in pMirGlo. Listed are sequences of UTR fragments; the 8mer that was mutated is underlined. The following mutations were made:

Original 8mer	Mutated 8mer
GAAGGTGA	cAAcGTcA
ACAGGGTA	AgAcGcTA
AGGTAAGT	tGGatAGT
GTACTATT	cTAgTATa
TGTTCTAT	TcTagTAT
GTTTATAT	GaTTAatT
GTGAGTTT	GaGtGTaT
GTTGCATT	GTacgtTT

PPP1R3C

CATTAATGGCTAACAACACGTAGGGACTTCATGTCATGTCAAAGATAGCTCTTTGCAAGTGCCTTGATTA
AACCAGAAAACTGTCATCGTTTAACCCAAATATCTGAATGGTCATCTGGTAACTCATGGGTTTTTGGCCT
CATAAGATGGTCCACTCTGTACACAGGCATTCCTCCTGCAATAATGTTGTATCTTTGAGACCGTTGTCAG
TGTACACAACTCACATCCTTCATATTGAAGGTGACTCATTTTTCTGCACACTTTTTTGATGTGATGCTTG
ACGTGAGGCCCGACACTAGGATTCTCAATGCAAGAATCCAGTACCTTGCACATAGAAGTAGCAACCCATC
CCTTGCCTATTTTCATCTTGCTGTTTTCTTTTTTTTAAAAAAATGGATGTGACTTGTTTTGAATGTTTTG
TATTATACTTGTTTTTGTGTGTGCATAAATTCATTCTGTAGGATCTTAAGAAAAAGAGTCCCAGAATGTT
GCTTCTATT

E2F1

TTATACCCCTCTCCTCTGTCTCCAGAAGCTTCTAGCTCTGGGGTCTGGCTACCGCTAGGAGGCTGAGCAA
GCCAGGAAGGGAAGGAGTCTGTGTGGTGTGTATGTGCATGCAGCCTACACCCACACGTGTGTACCGGGGG
TGAATGTGTGTGAGCATGTGTGTGTGCATGTACCGGGGAATGAAGGTGAACATACACCTCTGTGTGTGCA
CTGCAGACACGCCCCAGTGTGTCCACATGTGTGTGCATGAGTCCATGTGTGCGCGTGGGGGGGCTCTAAC
TGCACTTTCGGCCCTTTTGCTCTGGGGGTCCCACAAGGCCCAGGGCAGTGCCTGCTCCCAGAATCTGGTG
CTCTGACCAGGCCAGGTGGGGAGGCTTTGGCTGGCTGGGCGTGTAGGACGGTGAGAGCACTTCTGTCTTA
AAGGT

PRPF4B

AAAATTAATGCTTGTCTCCCTGAGGAATATGGAAAATAATCAGATTTTAGGGGTTTAAATAATATTTTTA
AATTGTAAATGGGATTTTTTTATTCACCCAGGCACCTAATTACAACAAGCATGCACATTTTGGTGCATTC
AAGAATGGAAAATCAGAATAGCAGCATTGATTCTTCTGGTGGGTTTTGCTCCATTTAAAGACATGAAATG
AACTACAGCCAGGAAGGTGATAGATGATATAATAAGCCACCTCTGAACCTACACCCCGTCTCTTCACGGT
TTAGACTTACTAAAATAAATACAAGGTGATTTTCATCTTCAGGTAGAGTGAAGCCTTTTAATTAAGGCGT
CACAGGTGCAGCTATTCTACCTTAATGAAATGGGTAGTGATTTTCCCACCATTATTTATTTCGGTGATAA
TATGCTGCATATTCAAGTCTCTTGTAGTTATTTTCACCCAAAGTAGTTGACAATTTGATGCTTCTGGTGA
TGTTTATGGCTTC

TRIM33

TTTTTCTCATTTTTATGCTGTTGGGTCTTAGTTTTTAAATTGATATAAAGAACTCAGCAATGGTTTTATT
TTCTACTCATACTTAGGGTTTAGGAAACACTACCACTAGTTATCATTTAATCAACTTCAATGGTCTACTG
AAACAAAAATGGTAACTTTTCATTAGTGGATTATTTAGAGTTATAGTAGTTGTTTCCAGAAAACACTTCC
TCACAATTGTACTTCCCAATCAAATCATGTGATCATACAGTTATTCCCATGAAAGGCAGAATGTTTGTTT
CAAAATTAATCTAGTTTTCTGTACATTTAAATTTGAGAAGGTGACAACTGGCTCTTTTCCAGTCTTCCTT

CATGTCAGTTTTCTGATAGACCACTATTGGCAAACAGTATCTGTCAACTACCAAATGTGTAAAATTTTCT
GTATTTCACTTTGTCTTATTTGTAAATAGTGAACTAAAACTTTTGGCAGATCAG

MTCH2

GAAAATAAGCAAGATTTGGAAAGGAAGTATAATGGCACTTTTTTCCTCAAAGGAAGTTCTTGTTTTCACA
TAAAATATGAAAAGCAGATCCTGCAGGAGTAACCCCCTTCTTTAAGAGCCAAGTATTTGCCAGTGCTTAA
ATTACACCATACAGTTCTAATTATATATAATCTTTTGTTCTTCAGTTTTTTGTTTTGTTTCCTTTTTGTT
ATTGTTGCCGAAGGTGAGTAGTTTTGCATTTCTGATGACAGCCTTGGAAAGTATATTTGTAACTCCATGT
CTGGTAATGCCAACCCAAGTCGACATGGGTCTTAGGACACTGACCACCTCACATGCCATACCCTCAGTTA
AGCATGTTAACATTTATAGGAGGAAAAAAATCACTTTGGGAGAAAATAAAATTCAACTCAAGCATAAAGC
TTCTGTTTACTCAGGCCTTCTAAAAAGCAGGTTAAAATGCTCTAAAATGAGAAAGCCTGTGGTTT

TBLX1R1

AGATACTGAAAGTGCCTCCTTTTGTGGTGTAAAAAACAAATTATGGTGCAAAAAGTAATCACTAGATTGA
AATACATGAAGGTTTTTTGCTTTTTGACATACGAAAATGTCAAGAGAAAGGCCAAAGATTTGTACTTTTT
CACTTACAAAGCACTCCTTTTTCCCTTAAACTTCTTTCTGTCAAATTAGATTTAATGAGAGAGTACTATT
TTTAAGGAGCTATCTGTTTATGTAGAATGATTTTGTTAAGAGTAATGTAAACTATTATTGAGTAGAGGCC
TAAAGAGGACTGTGCATTTTTGCTATTTAAAGGAATCACAAATGATCATACTTAAGTGAGCAAAAATGAC
AAGTTTTACTAGCTAAGTAGAGAAATAAATCTCAAATGCAGCGCTACAATTTTCATTATCTTAAGTACAT
TGTACATTTCTACAGAACCTGTGATTATTCTCGCATGATAAGGATGGTACTTGCATATGGTGAATT

VCAN

CTAACTTCCTGTGCCTTTCCTATCACCTCGAGAAGTAATTATCAGTTGGTTTGGATTTTTGGACCACCGT
TCAGTCATTTTGGGTTGCCGTGCTCCCAAAACATTTTAAATGAAAGTATTGGCATTCAAAAAGACAGCAG
ACAAAATGAAAGAAAATGAGAGCAGAAAGTAAGCATTTCCAGCCTATCTAATTTCTTTAGTTTTCTATTT
GCCTCCAGTGCAGTCCATTTCCTAATGTATACCAGCCTACTGTACTATTTAAAATGCTCAATTTCAGCAC
CGATGGCCATGTAAATAAGATGATTTAATGTTGATTTTAATCCTGTATATAAAATAAAAAGTCACAATGA
GTTTGGGCATATTTAATGATGATTATGGAGCCTTAGAGGTCTTTAATCATTGGTTCGGCTGCTTTTATGT
AGTTTAGGCTGGAAATGGTTTCACTTGCTCTTTGACTGTCAGCAAGACTGAAGATGGCTTTTCCTGGACA
GCTAGAAAACACAAAATCTTGTAGGTCATTGCACCTATCTCAGCCATAGGTGCAGTTTGCTTCTACAT

ARID2

GCATTATGTGCAGGTATGATATTTTCTTCACTACTTTTTCTATCTTAATATAGTGTGGAATTTTATTGTA
TTATTCTTCCATTCTTAATACTGTACCACATTCCTGCTCAGAAACTGCTCACTTCCTTAAATTGTCTTTT
TTCCCCCAGCGTGAAATGTATCCATTTATAACTGCCTATTGCCTGTTCTATTAGCATCCAAAAATGTGGA
AGGCCTCCCAACCACCATTTCTGCTGTGTCCTTAGGATGTGCAGTAAAAAATATAGACCTAACAGTTTAT
GTTATAGAATGGCTTTATTTACTTTGGTGACTGTTTATAGTTTTTAAATAAAAGACTGAACATTTTCTTG
AGTCCTTCATTTCTGAGTATGCTTAAGACATCTTAAAAATATAGAGAGAATTCTAAATTCAGCTGAAGGC
AAGGTATAACGGTCACCTACCTATTTGATTATATGTTGATTGATAACATATTAAATAGAGAACAAATAAG
AGAGGTCCTTTACATGACAAATTTGCATGAAATAAGCAGATTAACCAA

TGFBR1

CCCAGGACAGAAAATGTGTAGTCTACCTTTATTTTTTATTAACAAAACTTGTTTTTTAAAAAGATGATTG
CTGGTCTTAACTTTAGGTAACTCTGCTGTGCTGGAGATCATCTTTAAGGGCAAAGGAGTTGGATTGCTGA
ATTACAATGAAACATGTCTTATTACTAAAGAAAGTGATTTACTCCTGGTTAGTACATTCTCAGAGGATTC
TGAACCACTAGAGTTTCCTTGATTCAGACTTTGAATGTACTGTTCTATAGTTTTTCAGGATCTTAAAACT
AACACTTATAAAACTCTTATCTTGAGTCTAAAAATGACCTCATATAGTAGTGAGGAACATAATTCATGCA
ATTGTATTTTGTATACTATTATTGTTCTTTCACTTATTCAGAACATTACATGCCTTCAAAATGGGATTGT
ACTATACCAGTAAGTGCCACTTCTGTGTCTTTCTAATGGA

DCX

TAATTGTGACACCAGATGCTTAGGATCCTGGTGCTGGGTTAGCTAAGAGAATAGACAGAATTGGAAAATA
CTGCAGACATTTCCGAAGAGTTTATAAAGCACAGTGAATTCCTGGTCAATCTCTCCACTGAGGCAATTTG
GAATCAATAAGCAATTGATAATAGTTTGGAGTAAGGGACTTCATATACCTGATTCCTCTAGAAGGCTGTC
TAACATACCACATGATTACATGAACTGTATGGTATCCATCTATCTCTGTTCTATTGAATGCCTTGTTAAC
AGCCAACACTGAAAACACTGTGAGAATTTGTTTTCAGGTCTGACACCTTTCAGTCTCTTTTTATAGCAAG
AAATCAATATCCTTTTTATAAAAATTCATGTCTGTATTTCAGGAGCAAACTCTTCAGGCTCCTTTTTTAT
AAACTGGTGATTTTTCTTTTGTCTAAAAAACACATGAAGAAAATTTACCAAAAAAAAAAAAAAAAGCAGA
AGAATAATGTAGTTTAGAAATTATGCTGTCACTGCC

LPPR4

TTTCAGGTGCAGTATGATATTTCCTAATCTTTCCTATTTCTTAACAAAAGATTTTAAAGTACTTCTCTAG
TCATTGAAGTTTTTTTTTCTTTACATAAATATTGATATATTCTTTTTCTACTCAAAGTGCCAAAGGCTAC
AGTTTTTAATGACTTAACAAATTGTACCACATTGTTAAGGACATATAATGATAGACACTAGAACTCAGAC
CTCTGCATGTATATTTGATAACATGTCTTTTGTAAAACAAAAATTACAAAAAAATTTGTTTACATTCCAC
TGGTACCTTAATTTAAAATAAATCAGACTAAAAGGTGGTATCTCTTCTTAGTGTTCTATTTATCTTATTT
GCTAATGGGAGCACTTCTTCCTTTGTTAGGCTGTGCTTTACTGATAAAACCAAGTATTGAATAAAGAGAG
TTAATTATCTTTTTAAAGTAAATAAAATTATGAAAATATATATAGTATATATAAAGTACTGTGTTTAAAA
AAATGTTATGCAATGTTTTCCAAACTGATAAAG

ROBO1

ATACTACCCTTTGCTGGAAGAAACTAAAAATATAGCAAATGCAGAACCACAAACAATTCGAATGGGGTAG
AAACATTGTAAATATTTACTCTTTGCAAACCCTGGTGGTATTTTATTTTGGCTTCATTTCAATCATTGAA
GTATATTCTTATTGGAAATGTACTTTTGGATAAGTAGGGCTAAGCCAGTTGGATCTCTGGTTGTCTAGTC
ATTGTCATAAGTAAACCTAGTAAAACCTTGTTCTATTTTTCAATCATCAAAAAGTAATTATAAATACGTA
TTACAAACAAGTGGATGTTTTTAATGACCAATTGAGTAAGAACATCCCTGTCTTAACTGGCCTAAATTTC
TTCTGGTAGTGTCAGTTCAACTTTCAGAAGTGCCACTTAAGGAAGTTTGATTTTTGTTTTTGTAATGCAC
TGTTTTTAATCTCTCTCTCTTTTTTTTTTTTTTTTTGGTTTTAAAAGCACAATCACTAAACTTTATTTGT
AAACCATTGTAACTATTAACCTTTTTTGTCTTATTGAAAAAAAAAAATGTTGAGAAGCGTTTTTAACCTGT
TTTGTT

ETS1

AGTTTTGAAGCAAAGATGGACTTCAGTGGGGAGGGGCCAAAACCGTTGTTGTGTTAAAATTTATTTTATT
AAATTTTGTGCCAGTATTTTTTTTCTTAAAAATCGTCTTAAGCTCTAAGGTGGTCTCAGTATTGCAATAT
CATGTAAGTTTGTTTTTATTTGCCGGCTGAGGATTCTGTCACAATGAAAGAAAACTGTTTATATAGACCC
CATTGGAAAAGCAAAACGCTCTCACTGAGATCAGGGATCCCAAATTCATGGGACTTATATAAGAAGGACA
ATTAATGCTGATTTGGGTACAGGGGAATTATGTGTGTGAATGTCATCTACAATTAAAAAAAATTAGCACA
TCCCTTTACTTACTTGTTATCAGTGGATTCTCGGGGTTTGGACTTAATGTTGAGCTAAGAAGCATTAAGT
CTTTGAACTGAATGTATTTTGCATCCCTGGTTTTGGACGACAGTAAACGTAGGAGCACTGTTGAAG

BCOR

AAATGAGTAAAAGGAGCTCCACACTTTGACTTAATTTCATACAAAGCTCTGATGACAGGCCATGACTGTA
GAGTGGTCAGAACTGTGTGGTTGGTTTGAGGGAGCGAATTCGGGGAAGGCACTTGGTGATATAACTTTGT
TTTGTTTACAGAGTACCTGCTCGGGCCAGGTAAATGCTATTGGATGTAATCCAGTAGTGTGTAATATAAA
TTCAAACCATATCCACACACAACAACTAATTGTATGAAACTTTTATATCCTAATTTAAAAGCTGTGAAAT
TAGTTTTCACGCATCAAACCGGATTGTTTATATGTTTAAACATTTTATGCTCTTATTTAAAGAAGACTTT
GAGCTATTTTTTTCTGTACCCTGTAAAATATTGAAAACTAACATAATATGTTGAGGTTGCTTGGAAATGT
ACATAAAACTAAAATTTTCTGAATCGTGTGTTTATGTTTGAA

RAB1A

AACAAAATTGCCTGAATTGTACTGTATGTAGCTGCACTACAACAGATTCTTACCGTCTCCACAAAGGTCA
GAGATTGTAAATGGTCAATACTGACTTTTTTTTTATTCCCTTGACTCAAGACAGCTAACTTCATTTTCAG
AACTGTTTTAAACCTTTGTGTGCTGGTTTATAAAATAATGTGTGTAATCCTTGTTGCTTTCCTGATACCA
GACTGTTTCCCGTGGTTGGTTAGAATATATTTTGTTTTGATGTTTATATTGGCATGTTTAGATGTCAGGT
TTAGTCTTCTGAAGATGAAGTTCAGCCATTTTGTATCAAACAGCACAAGCAGTGTCTGTCACTTTCCATG
CATAAAGTTTAGTGAGATGTTATATGTAAGATCTGATTTGCTAGTTCTTCCTTGTAGAGTTATAAATGGA
AAGATTACACTATCTGATTAATAGTTTCTTCATACTCTGCATATAATTTGTGGCTGCAGAAT

GTF2H1

ATGCTATTGTACTTGCACATTGGAGACTGAAAGGAAAGAAGGGACTAAATGCTGGGGAGGTAAATTAAGA
CAGAACCAAATGAGCTAAGTTGCAAATATATATATATACACACACACACATATATGTACATGTGTATGTA
CATATATATTTTAAAAGACTGTTTACTGCAGTTGCTCAGGAACTGCTTTTGATTCACATTAAGCTGCTTT
CAGAAATTAAAAAAACACTTTTTAAAGGGTGCATTGATAAAATCTGAGGTTTTTTGGTTGTCGTTTTTTT
CTGTGTACATTTTTTTCCTAAGTTTATGGCACAGGGTAGACCTTAAGTATTCCTCCTCCATCCTTCATTC
TTCACCCTCCATTGGATCCTCAAGTTTTAATGAATTCCAATTATACCTTACATCAGCAAGTTAAAAAAAG
TACTTTAAAATAAAGCAAAGGGAGACTGTTGCTCAACCATCAGGAAACAGTTGTCAGAAGA

HOXA3

CCTTTCCTTCCTTCCTTTTCTCTCTTCTCCGCCCCGCACTCCGTTTCCCGGTTTCCCCCCTCGTTGGTAA
GGCGTTTTTATAGTTTATGTGACGTAGCAATCTTGGTTGCTGGAATGGCTGTATCATAGCGATATTTATC
TCTTCCTGCTCCTCGATAGGCCACTGGCCCTGCACCCTTTACCTTCTCCACTCTTTGATCAGAAACAGGG
TATATGAACAAATTTTCTAGTCGAGTTTTCAATGTGAATTTGTTCTTACATTATGGCTCCCGAGGGGAAG
CGATTACTTTTTTTAATTTTAAATTTTTTTTTTTAATTGCACTTCTTGTAAAGAGTGAGAAAAAAAAATCAA
AGGCGCTTTGAAACAGGGGCTCTCTGTGCAAGGATGACTAAGTGTACGTCTTTCCGTGTGTGTATGCTGG
TGAACAGTCAGATTTATTTATATTTTTTTGCAAGCATTGAATAATCTAAGTTTTAAATATTATTTATCCC
CATCCGTTCGTATTTATATTAAAGAATTCTGTACCCTGATGGTTCAGAAGGGTTCTTGGGCCTTTTGTTC
AATTGTGTATT

MAP3K7

CAGAACAGGAGGTATCAAACTAGCTGCTATGTGCAAACAGCGTCCATTTTTTCATATTAGAGGTGGAACC
TCAAGAATGACTTTATTCTTGTATCTCATCTCAAAATATTAATAATTTTTTTCCCAAAAGATGGTATATA
CCAAGTTAAAGACAGGGTATTATAAATTTAGAGTGATTGGTGGTATATTACGGAAATACGGAACCTTTAG
GGATAGTTCCGTGTAAGGGCTTTGATGCCAGCATCCTTGGATCAGTACTGAACTCAGTTCCATCCGTAAA
ATATGTAAAGGTAAGTGGCAGCTGCTCTATTTAATGAAAGCAGTTTTACCGGATTTTGTTAGACTAAAAT
TTGATTGTGATACATTGAACAAAATGGAACTCATTTTTTTTTAAGGAGTAAAGATTTTTAATTCTGTGAT
TGTGTGTATGTGTGTTGAAACTGTAAAGCTTTTATGACTCTAATATTAATCTCTTAAATGAAATTAAAAG
GCAAAAGAACATGATTGAGC

TBX5

AATGATATCTCGCCAAAGAAACCACGCCCACACCAATGCCAACACAAAACTGTGTTTACTGAAAGCCGAA
AACAGTATTAAAAAAAGTGTGTAAGTAAAGTGTTATGGTAGGGTTCTTCAGATGTAATATTTTACTGGTA
CTATTTATTTATAAATAGGAATTCTAATTAAGTAATAACATGAAATGAAACCCAGCATAGGAGCTGGCCA
AGAGCTTTTAATTTTATTGATACTCAAAACCAAGTTTGTGTTTTTTTGTTTTTTTTTGTTTTTTTCCTCT
TTCGAATGTGCTTTGCTTTTTTTGATTAAAAAGAATTTTTTTTTTCCTTTTTTATAAACAGACCCTAATA
AAGAGAACAGGGTAAGATGTGAGGCTGAGTGTGTTTAAGTACGTGAGAGAGTGTGAGTGTGTTTGTAAGT
GAGTGTCCCTATGCGATTATGTCTCTTTACGTTGCTAAGGG

TFAP2C

TGGGAGACTCTTTGAAATGACATGTTCCTTTAAGGTACTGAAGCTTTATTTGCATATTTATTTCAGATGT
TTCGAGTAAACTTGAAAAGGGTAGGCACGAAGCAATTTGTTGCTGCTTGTCACCCCCAAGTCCCCGTGGA

GGTTCTGTATTTTAAGAAACAGTGCGTTGAGTGTACAGATTTTATTTATGCGTAATTTAATGGGGTCTGT
AAATACTGGTGCACTTCTTACGACTTTTTTGAGACATGGGATCCAATTTTAATATTAACTTTTAATGGTG
ATGGGGTAATCTATAACACATCATAAGGTTTTATTCATATATATACAGGGTATTAAGAATTAAGAGGATG
CTGGGCTCTGTTCTTGGCTTGGAAGATTCTATTTAATTGAAACTCTCTGTTCAGAAAGCAATAACTTTGT
CTCGTTCCTGTTGGGCTGAACCCTAAGGTGAGTGTGCAGTACAGTGTGTGTGGGTGAAATGGAGATTTGG
AATTGAACTCTCTGCCTGTAAATGTTC

DST
AAGAGGCGAGTTTAAAATTCTGCAGATGGCCTTATTTGTGTATTTGTCTTTTTATTTTATCTGTATAATT
TTTTTTGTCAGATATTCTGGGGTTAAAGTCACATCATATGTGAGGAGGAAAAGTTTAACATGAACTAACA
TTTCTGCACTGTAACGTGCCGGGCACACACTAAACTCAGTTACTGTACCTACAGGTAAGTCTACATCCTC
TCTGACAGCCACAGCACTACATCAATCCCTGACGTTAGGGATACCTCATGACATTTTCCTGTTTTTATGG
AAACTCTGAGAAGCTGAATGATACATGCAGGGGATATTTTTTGAGATGATTTAAATGTAAACCAAAAGAT
GGAAGACAAAAAGACAAACACACCCACACGCAGTCTTTGCAGTATCTGACAGAGAACTCACAGGAAGTTA
CTTCAAGCACTTGCCAGTACTATGATATTCAAGTACCTTGCAGCATTTCTCTGCCATTGCTTTCAATGAG
GCCAGAGGCATCCTGGATATTAGACCTATTATACTGTAAGAATATAAGTATAAAGTGCGTTCATATACAT
GTGAGGTTTTCTTTTGCTTGAGTG

GABRB3
GTCAGTGTGAGCCATTAGAGTAACATCGAATCTTGGGGCAAAGAACTGCCCAGGTGAATTAAATTTTTCC
AGGACACTAGCTAGTGTGCCTTGGATTGATTACCTCTTCTACTGCATTGAAAGGCGCCATGTTTTCCTGA
AATACTAAATTCCCAACACCTGGGTAAACAATGACCTTCCAGAGAGTGGCTCCCGTATGCCTCTCCCTAG
GACCAACCCCATGAACATGTTTTGTCACGTTTGTCTCATGTTTCTACTTCACAAGTCAGTGAGTGTGTTT
AAGGTAAGTACAGGATTATTCTAGTAGGAATAGGCGATTGCTGTCATAATCAATTCTCATGTTGATTTCA
TTTTATTGTAAAGATAAATTTAAACCCAGTTTTGCTTAAGCACATTGATGTAATTTTTTGGTATTATTTG
ACATGAAAAAACAGCAAAATTGAGTGATAGATACA

RPS6KB1
TGCTGTGGAAAGTTTATTGAGAACTTGTTTCATAAATGGATATCCCTACTATGACTGTGAAAACATGTCA
AGTGTCACATTAGTGTCACAGACAGAAAGCACACACCTATGCAATATGGCTTATCTATATTTATTTGTAA
AAATCCAAGCATAGTTTAAAATATGATGTCGATATTACTAGTCTTGAGTTTCTAAGAGGGTTCTTTATGT
TATACCAGGTAAGTGTATAAAAGAGATTAAGTGCTTTTTTTTCATCACTTGATTATTTTCTTTAAAATCA
GCTATTACAGGATATTTTTTTATTTTATACATGCTGTTTTTTAATTAAAATATAATCACTGAAGTTTACT
AATTTGATTTTATAAGGTTTGTAGCATTACAGAATAACTAAACTGGGATTTATAAACCAGCTGTGATTAA
CAATGTAAAGTATTAATTATTGAACTTTGAACCAGATTTTTAGGAAAATTATGTTCTTTTTCCCCCTTTA
TGGTCTTAACTAATTTGAATCCTTCAAGAAGGATTTTTCCATACTATTTTTTAAGATAGAAGATAATTTG
TGGGCAGG

SBK1
ATTTGAGGGGCTCTTTGATGGGCCAGGCCGGCCAGAGTGAACTCCGAGCACTTTCTGGCTGGTGCCCCAA
CCTCTCCACTCCCCACTCATTCCCACCTTGAAAAAGGGCTATAGGTCCCCTGCCCTGCCCGGGTCCAGTT
TACAAACAGTGTGGGGTGGCCCCAGGGCCTGGCCCCACTCTCCCTGCTGTGCCCACTCCTCTCCAGACTC
CACCTCCCCAGTGGGTATGGGCCCTCCACATGCCAGGTAAGTAGCAAACCCCCACTCCCTCCAAGGACCA
GGTCTCAGAGAAGGCCCTGGTCACTGCCCCCGGCCCACCTGGAGCCCATCGGGGCTGCCTCTCCCAGCCG
CGACTTCTCCTTTTGCCTTAGGCCTCGCGACATCCTGATCTCTCCTGCAATAACTAGGAATCGAGATTCC
ACAGTAGACGTCCCTTGCCGTGCTCGCTCTCTCTCTCGCGCGCGCTCTCTCTCTCCCTCTCTCTCTCTCT
CTCTCTCTCTCTCTCTCTCTCTCTCTCTCTCTCTCTCCTCTCTTTCTCTCTCTCCCTCTCTCTGTTAAGATC
CTGTTCGGGAGTTTCCCCAGCCGTTGTAGTATCTAGTAT

ARID1A

AAACCACCTCAGAATCCAGTTTACCCTGTGCTGTCCAGCTTCTCCCTTGGGAAAAAGTCTCTCCTGTTTC
TCTCTCCTCCTTCCACCTCCCCTCCCTCCATCACCTCACGCCTTTCTGTTCCTTGTCCTCACCTTACTCC
CCTCAGGACCCTACCCCACCCTCTTTGAAAAGACAAAGCTCTGCCTACATAGAAGACTTTTTTTATTTTA
ACCAAAGTTACTGTTGTTTACAGTGAGTTTGGGGAAAAAAAAATAAAATAAAAATGGCTTTCCCAGTCCTT
GCATCAACGGGATGCCACATTTCATAACTGTTTTTAATGGTAAAAAAAAAAAAAAAAAAATACAAAAAAAA
ATTCTGAAGGACAAAAAAGGTGACTGCTGAACTGTGTGTGGTTTATTGTTGTACATTCACAATCTTGCAG
GAGCCAAGAAGTTCGCAGTTGTGAACAGACCCTGTTCACTGGAGAGGCCTGTGCAGTAGAGTGTAGACCC
TTTCATGTACTGT

NFAT5

TGATTGAGAGGCATTGAATTACGTTTTCAGTAGTACAGGCTTCTTGCCGATATGAAGGGAACTTTTCAGA
AAGAGACCTACTCTGGGTCATTTAATTTTGAATACAGTTTTCAATCGTTCAAGTTTTGGATGGTTTATAT
CTAATGTGTGTTTCATTTTTTTGGAAAGCTATATTTTGTATTTAGGAAATGGTATACTATTTTGCTATTT
GTACTGAGTGAGTACATTGGCATAAATATAGAAATTTATATATATACATATATATAAACTATTCTTTTTT
GCCACACATTTTTGTGGTAAATTTGTGAGTTTGTCTGATGTTCTACCACAACGTGGCGTCTGATAACAGT
GAGGGGGGGTGGGGTTTGTTATGTCTTTATTGAGTATTTAAGTATCTTTTGAAACAAATGACCTGTTCAT
CTGTGGCCATTCCATCAGGCAGTTAGTTCCTTGATGTCAGTAGTGGGCTAAAGGCAGCTTACTGTGTGTT
TGCTGGAGCTTTCACTCAGCCAAGTGTTAGAGTCAGGAAACCCATTGAGGCAATGGCGTCAAATGGTGTT
TCACAAGAATGAGCCATTCAGTCTTTGCTCACTATATATTTAATATTTTATTATTGTTGTTATTGTTATT
ATTAATTGGCTTTCTGTATTCTATGCCTTTT

RYBP

ATACCACGCAATCTTTCACACTCGTGCGTGTGCGCGCACACAGAGCTTACCTGACTTGCTCTGCTTGAGT
CATGCAGTTACAAAAAAAAAGACATCTTGACACCCACACAATATTCTAATCAAAACCTTTCAGTTTCAAT
CTGGATATTTAAAAACATTGGCAGAAGCTTCTGTGAGTTTAGTTCCACTAAGATGTTTCACCTGCCTTAT
CAAGACCATTCTCAGTCTACTTTTTTAAGCTACCGTATCTTAAATTATTGAAAATTTATTAATTGCTGAA
TATATAATAACCTTTGCTTGTATGTAACCGAAAATGGTTTAAGAGCCAACATTTAGAGTATGACAATGGA
GCTGAACAGTTTTTAATGCGCAAGCAGTTCTGTTCTTGTGTATGACTTGTAACCTTAATTTACTGTGTAA
AGATGGTTACATTATTTCCTTAGCTTTGTTTGTTGGAGACAAATAGAGAATGCTTGTTAAGTATGTCAAA
ACAATCTTATCTTGTGAATTTTTGTTAATGTATTATACGAGCTATATTTTTCATTTGCCCAGAAAGACAG
CTTGTATAACGCTTTTGGAAG

SOX11

GGTTGATCTGGAAAATTCTGCTCTTTGATAACAACTCAGGATTTTTTTGTTCAGTTTTGGTTTTTGCCCC
TTCCTGTGGAGCCTACATTTTCAACCACAATAAAGATGAAACAAAATTTATGAAACTGAGCTCTCTTCCA
TTTTACTTACTGCTGGCTTTTTTTTTTTTTCCTTGATTCCTACCATACCTTCGTTTTTTTCATTGTACTT
TTTTAACACTACCTATATCCATTAGCTGCCTAATTAGTTTTATCTGTTCCATGTGGATGCAGTGAGTTTA
TAAGAGAATTTCACAAACAAGTAGTTTTTTAGTGAACTTAAAATAAACAGAATTTTAAAGGAGACCTATT
TTTATACTCAATAAAAGCACAAAAGTGCAGAAAGTATAAAACGGCTTACAAAGGGAGACACAAGCTCATA
ATGTTCCATGTATAAAAGTAATAACTTTATTGGGTAGAGATATTCTTACAAGATCTAGCACCTCTGCCAG
TGCACAGATAGGACTGTTTTAAATGATTTGGGAACTTTTGGTTGCCTGCAGTTGTGAACAGAGAACTTCT
CTACAGAGAAACAAACCACTAAAAGCAATATGACCGAGT

SYNJ1

CCACCTAAATTCCTATGTCCACACTTAACCTTTAAAGATGTACATTGAGGGAATATCAAAAAATAGCGTT
CATGGCTACGAATATGTAGAATGTTAAAAGCACAGCAAACTGCACTGCACTTATAAAGCAAATCTATTAG
AAAAAAAGCATTTTTCTAAATGCTCAAATGTTATCAAAATACTATATTTATAGAAGTAATCTTCTCTGTT
AACAGCCAAGATTTGCTGTTAGAAATAACTCTTGTGAGTTTTATATTGTGCTTTTTGGAGGTTCTAATCA
TTTCAGCAGTAGCGTCTTAAACAGCAGTATTACTATAAGCAGTTGCTTCAAAATGTGAATTAACTTGTTG
AAACTGTGGCTTTAACATCCATGTGACTAGTGTATATGGTATTTGCTCTCCATTAGCAAAATAATTCATT

GTTAGGTAAACTTCACTAGTGCAAATTGCAGATCTGTGAGCAATGTTTCCTATTGAATATAAACTACTGT
CTAAAATATACATATCAGCAGCCCAGC

ETV1

CAGTCAAGCAGGGCGTTTTTGCGCTTTTCCTTTTTTCTGCAAGATACAGAGAATTGCTGAATCTTTGTTT
TATTTCTGTTGTTTGTATTTTATTTTTAAATAATAATACACAAAAAGGGGCTTTTCCTGTTGCATTATTC
TATGGTCTGCCATGGACTGTGCACTTTATTTGAGGGTGGGTGGGAGTAATCTAAACATTTATTCTGTGTA
ACAGGAAGCTAATGGGTGAATGGGCAGAGGGATTTGGGGATTACTTTTTACTTAGGCTTGGGATGGGGTC
CTACAAGTTTTGAGTATGATGAAACTATATCATGTCTGTTTGATTTCATAACAACATAAGATAATGTTTA
TTTTATCGGGGTATCTATGGTACAGTTAATTTCACGTTGTGTAAATATCCACTTGGAGACTATTTGCCTT
GGGCATTTTCCCCTGTCATTTATGAGTCTCTGCAGGTGTACAAAAAAACCCCAATCTACTGTAAATGGCA
G

FNDC3A

CTCACTCTGTCTGGTATAGGCTAATTTTGAAGAACTCCCATAAGTTTCTGCTGCTTCTCCCATAACTGCT
GCCACCACCATCAGAATTCATAATCAAACCTAACCTTTTTGTTTGGGGCACCAAATCTGAAGACAAAATT
AATTTGCACCAGTAAACTTCAAGCTGCTTTCTTTCTTGAAAACTAAACGTTTAACGTATAATGTCTGTTT
GGATACTGTTCCAAATTGTTGATTGCATGTGGTTAATGTTGCATTAGAGCACTTTGCAATTGCATAATTC
ATTAATGTTTTGTGAGCTTGCATTTGTGAGTTATTGGATGATCAGACTGAATTTTGTCAAGTATCACATT
GTACATCTTGCCTAGATGTCGATGACTGCAAGTAATAATACAGTTTATAATGAAACTATCTACAATTCTT
GTTTTAGCACATCTGTTATCCGTAAAACACCTGTAACTAGCTT

FZD5

GGGCGTTTTGTTTGGTAGTTTTGCCAAGGTCACTTCCGTTTACCTTCATGGTGCTGTTGCCCCCTCCCGC
GGCGACTTGGAGAGAGGGAAGAGGGGCGTTTTCGAGGAAGAACCTGTCCCAGGTCTTCTCCAAGGGGCCC
AGCTCACGTGTATTCTATTTTGCGTTTCTTACTGCCTTCTTTATGGGAACCCTCTTTTTAATTTATATGT
ATTTTTCTTAATTTGTAACTTTGTTGCATTTTGGCAACAATTTACCTTTGCTTTGGGGGCTTTACAATCC
TAAGGTTGGCGTTGTAATGAAGTTCCACTTGGTTCAGGTTTCTTTGAACTGTGTGGTCTCAATTGGGAAA
ATATATTTCCTATACGTGTGTCTTTAAAAAAAAATGTGAACAGTGAACGTTTCGGTTGCTGTGACTGGGA
AGTTGTTGGGTGTGCTTTTTCAGCCAGCTTCTCCTTCCACTGCTTAAAGTGTCCATGATTCTTTAAGGTG
AGCTGCAGTT

ROCK1

CCCTCACTGTAGAATTTAAAAGCCTTACTGTTGATTGCCCATGGTGGACTTGATGGAGAAATTAAATATC
TTTCATTATGCTTTACAAAATACTGTATATGTTTCAGCAAGTTTGGGGAATGGGAGAGGACGAAAAAAAG
TTACATTTAATCTATGCATTTTTGCCAAGCCATATTGAGTTATTTTACTACTAGAGACATTAGGAAACTA
ACTGTACAAAAGAACCAAGTTTAAAAGCATTTTGTGGGGTACATCATTTCTATAATTGTATAATGTATTT
CTTTGTGGTTTTAAATGATAAAGACATTAAGTTAACAAACATATAAGAAATGTATGCACTGTTTGAAATG
TAAATTATTCTTAGAACACTTTCAATGGGGGTTGCATTGTCCTTTTAGTGCCTTAATTTGAGATAATTAT
TTTACTGCCATGAGTAAGTATAGAAATTTCAAAAAATGTATTTTCAAAAAATTATGTGTGTCAGTGAGTT
TTTCATTGATAATTGGTTTAACTTAAAATATTTAGAGGTTTGTTGGACTTTCATAAATTGAGTACAATCT
TTGCATCAAACTACCTGCTACAATAATGACTTTATAAAACTGCAAAAAATGTAGAAGGTTGCACCAACAT
AAAAAGGAAATATGGCAATACATCTATGA

WNT5A

CTTTGGTTGTAGGACAGGAAATGAAACATTAGGAGCTCTGCTTGGAAAACAGTTCACTACTTAGGGATTT
TTGTTTCCTAAAACTTTTATTTTGAGGAGCAGTAGTTTTCTATGTTTTAATGACAGAACTTGGCTAATGG
AATTCACAGAGGTGTTGCAGCGTATCACTGTTATGATCCTGTGTTTAGATTATCCACTCATGCTTCTCCT
ATTGTACTGCAGGTGTACCTTAAAACTGTTCCCAGTGTACTTGAACAGTTGCATTTATAAGGGGGGAAAT
GTGGTTTAATGGTGCCTGATATCTCAAAGTCTTTTGTACATAACATATATATATATATACATATATATAA
ATATAAATATAAATATATCTCATTGCAGCCAGTGATTTAGATTTACAGTTTACTCTGGGGTTATTTCTCT

GTCTAGAGCATTGTTGTCCTTCACTGCAGTCCAGTTGGGATTATTCCAAAAGTTTTTTGAGTCTTGAGCT
TGGGCTGTGGCCCTGCTGTGATCATACCTTGAGCACGACGAAGCAACCTTGTTTCTGAGGAAGCTTGAGT
TCTG
